# N_2_ cleavage by silylene and formation of H_2_Si(*μ*-N)_2_SiH_2_

**DOI:** 10.1038/s41467-024-48064-z

**Published:** 2024-05-08

**Authors:** Liyan Cai, Bing Xu, Juanjuan Cheng, Fei Cong, Sebastian Riedel, Xuefeng Wang

**Affiliations:** 1 School of Chemical Science and Engineering, Shanghai Key Lab of Chemical Assessment and Sustainability, Tongji University, Shanghai, 200092 China; 2 Institut für Chemie und Biochemie – Anorganische Chemie, Freie Universität Berlin, Fabeckstrasse 34-36, D-14195 Berlin, Germany

**Keywords:** Reaction mechanisms, Structure elucidation, Chemical bonding

## Abstract

Fixation and functionalisation of N_2_ by main-group elements has remained scarce. Herein, we report a fixation and cleavage of the N ≡ N triple bond achieved in a dinitrogen (N_2_) matrix by the reaction of hydrogen and laser-ablated silicon atoms. The four-membered heterocycle H_2_Si(*μ*-N)_2_SiH_2_, the H_2_SiNN(H_2_) and HNSiNH complexes are characterized by infrared spectroscopy in conjunction with quantum-chemical calculations. The synergistic interaction of the two SiH_2_ moieties with N_2_ results in the formation of final product H_2_Si(*μ*-N)_2_SiH_2_, and theoretical calculations reveal the donation of electron density of Si to π* antibonding orbitals and the removal of electron density from the π bonding orbitals of N_2_, leading to cleave the non-polar and strong NN triple bond.

## Abstract (web summary)

So far, only homoleptic N_2_ complexes of silicon have been reported under matrix-isolation conditions including but the activation of more inert nitrogen by silylenes remains challenging. Here the authors report on the activation of dinitrogen by silylenes under cryogenic conditions.

## Introduction

**Fig. 1:**
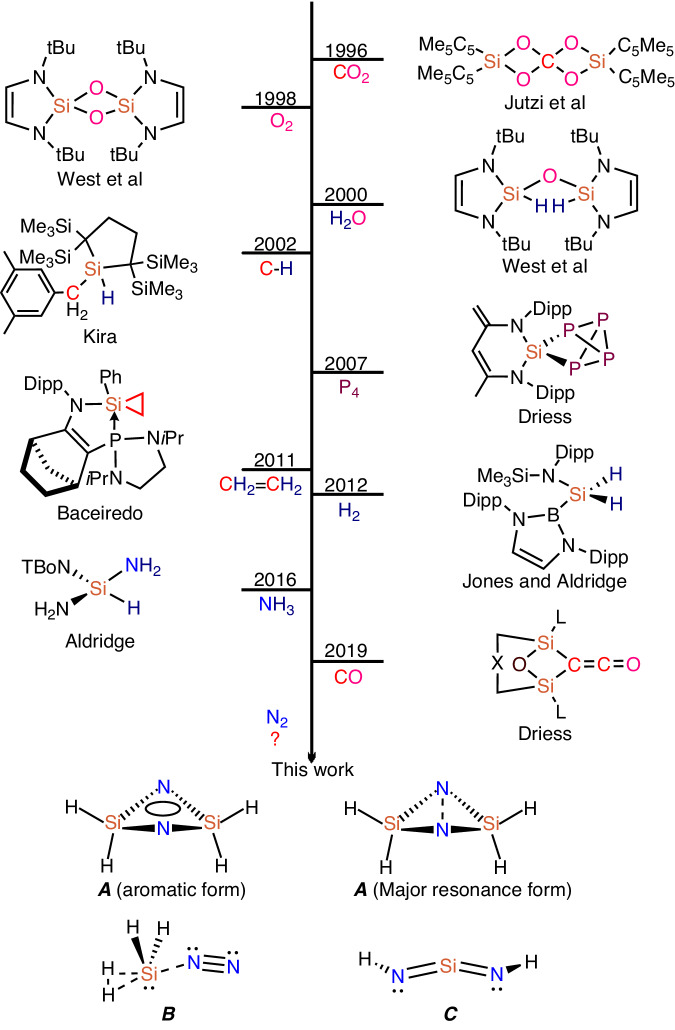
Binding modes of small molecules to silylene. (Dipp = 2,6-^i^Pr_2_C_6_H_3_; TBoN = N(SiMe_3_){B(DippNCH)_2_}; L = PhC(N*t*Bu)_2_; X = 9, 9-dimethyl-xanthene- 4, 5-diyl or 1, 1′ - ferrocenyl).

The industrial synthesis of NH_3_ relies on the transition metal-catalyzed Haber-Bosch process^1–3^, in which the inert dinitrogen is converted to ammonia under harsh reaction conditions. This N≡N triple bond activation is based on partially filled *d*-orbitals of the d-block elements (e.g., Fe), which have a suitable symmetry and energy. An alternative way to activate dinitrogen is through main-group element compounds via the π back-donation from either the *d* orbitals (Ca, Sr, Ba) or *p* orbitals (Be, B, C)^4^. Wherein *p*-block elements (e.g., B and C) have a non-bonding (donor) electron pair and an energetically low-lying vacant (acceptor) orbital which can mimic the *d*-orbital character to activate dinitrogen^5–7^. Recently the *p*-block element boron was successfully used to activate dinitrogen^8–10^. Five- and seven-electron boron-centered radicals (R_2_B^•^) are predicted to be favorable for dinitrogen activation both thermodynamically and kinetically^11,12^. A crucial metric for nitrogen activation of substantially elongated N–N bond has been achieved by a borylene coordinating an N_2_ molecule in an end-on bridging position as [{(CAAC)-DurB}_2_(μ^2^-N_2_)] (CAAC = cyclic alkylamino carbene, Dur = 2,3,5,6-tetramethylphenyl)^13–15^. Our groups reported a complete cleavage of the N≡N triple bond by fluoroborylene (:BF) which has been observed as a cyclic FB(*μ*-N)_2_BF system in a matrix-isolation investigation under cryogenic conditions^16^. Carbene, another reactive intermediate, has also been used for N_2_ activation and conversion^17–19^. Maier et al. found that singlet σ^0^π^2^ carbene (2-diazo-2H-imidazole) would bind with dinitrogen in the matrix, demonstrating the potential for σ^0^π^2^ carbene to activate dinitrogen^20^. Furthermore, N_2_ activation by a carbene pair has been calculated and the N≡N triple bond was predicted to be elongated to an N-N single bond (1.428 Å) under the synergistic effect of the two CH_2_ moieties^21^. Silylene, the silicon analog of carbene, has been proven to exhibit similar properties to transition metal compounds, as it has a narrow HOMO-LUMO energy gap, which has received particular attention for the activation of small molecules^22^. As reported in 1998, the first isolable N-heterocyclic silylene reacts rapidly with dry O_2_, giving rise to a colorless and insoluble disiladioxetane polymer^23^. From then on, many small stable molecules, such as CO_2_^24^, H_2_O^25^, P_4_^26^, C_2_H_4_^27^, H_2_^28^, NH_3_^29^, and C-H bonds^30^ have been activated by silylenes (see Fig. 1). In 2019 the splitting and reductive homocoupling of CO was achieved using the bis-silylenes (LSi:)_2_Xant [Xant = 9,9-dimethylxanthene-4,5-diyl; L = PhC(NtBu)_2_] and (LSi:)_2_Fc (Fc = 1,10-ferrocenyl)^31^. So far, only homoleptic N_2_ complexes of silicon have been reported under matrix-isolation conditions, including SiNN, Si(NN)_2_^32^, and larger silicon-nitrogen clusters^33,34^, but the activation of more inert nitrogen by silylenes remain a challenge, although silylenes do exhibit high reactivity for other small molecules^24–31^. The key difficulty in silylene-mediated nitrogen activation is to modify the occupied and vacant orbitals of silylene in space and energy, which could enhance the weakening and functionalization of an inert chemical bond. For example, Driess et al. reported that two silylene moieties (bis-silylenes) could be cooperative in cleaving unreactive bonds, in which the Si---Si distance plays a crucial role^31,35^.

Here we report on the activation of dinitrogen by silylenes under cryogenic conditions. In our matrix-isolation experiments, laser-ablated silicon atoms have been reacted with H_2_, D_2_, HD, and H_2_/D_2_ mixtures in solid nitrogen that served as both reactant and host-matrix material. H_2_Si(*μ*-N)_2_SiH_2_ (**A**) is spectroscopically and quantum-chemically characterized, which demonstrates that the N_2_ triple bond is cleaved by the synergistic interaction of the two SiH_2_ moieties. In addition H_2_SiNN(H_2_) (**B**) and HNSiNH (**C**) are identified.

## Results

**Fig. 2:**
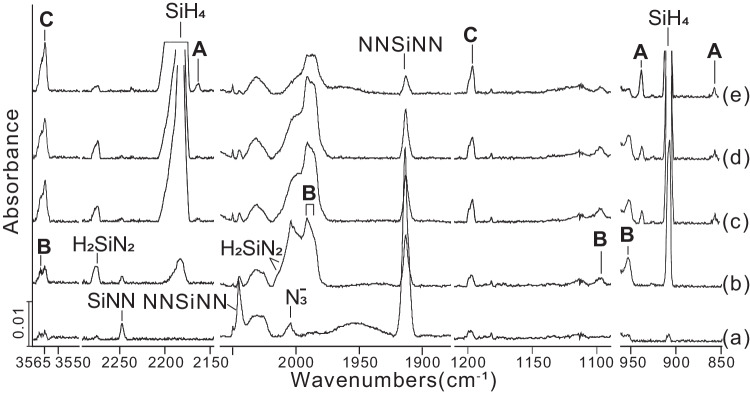
Infrared spectra of the reaction products of laser-ablated Si atoms with hydrogen under an excess of solid nitrogen at 4 K. **a** Codeposition of Si + 10% H_2_ for 120 min; **b** after λ > 300 nm irradiation for 10 min; **c** after λ > 220 nm irradiation for 10 min; **d** after annealing to 7 K; **e** after λ > 220 nm irradiation for 10 min. A H_2_Si(*μ*-N)_2_SiH_2_, B H_2_SiNN(H_2_), C HNSiNH.

**Fig. 3:**
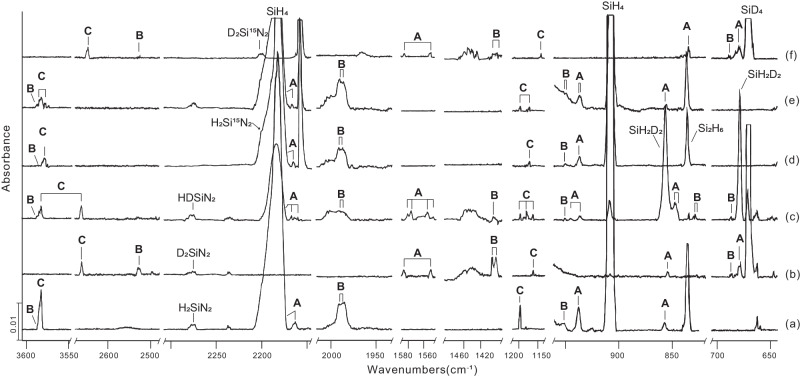
Infrared spectra of the laser-ablated Si atoms reactions with H_2_, D_2_ and HD in excess solid ^14^N_2_ (^15^N_2_) at 4 K after the second λ > 220 nm irradiation for 10 min. **a** Si + 10% H_2_/^14^N^14^N; (**b**) Si + 10% D_2_/^14^N^14^N; **c** Si + 15% HD/^14^N^14^N; **d** Si + 10% H_2_/^15^N^15^N; **e** Si + 10% H_2_/^14^N^14^N + ^15^N^15^N; **f** Si + 10% D_2_/^15^N^15^N. **A** H_2_Si(*μ*-N)_2_SiH_2_, **B** H_2_SiNN(H_2_), **C** HNSiNH.

Figures 2 and 3 show infrared spectra obtained after the laser-ablation of Si atoms co-deposited with a 10 % H_2_/N_2_ mixture at 4 K in a dinitrogen matrix. Further details are provided in the Supplementary Information (Supplementary Figs. 1–6). Isotopic experiments with H_2_, D_2_, HD, and H_2_/D_2_ samples in pure ^14^N_2_, ^15^N_2_, and ^14/15^N_2_ mixtures together with frequency calculations at the DFT level were used for the product identification (see Table 1 and Supplementary Tables 1–6). The EDA-NOCV method was used to elucidate the peculiar stability of the bonding nature. In addition to the three adduct products **A,** **B,** and **C**, H_2_SiNN, Si(NN)_2_, and SiNN have been observed (Fig. 1 and Supplementary Tables 1, 7)^32,36,37^. The absorptions associated with three silicon-nitrogen hydrides H_2_Si(*μ*-N)_2_SiH_2_ (**A**), H_2_SiNN(H_2_) (**B**), and HNSiNH (**C**) were unambiguously assigned based on their growth/decay behavior in different experiments and on their characteristic H/D and ^14/15^N isotope pattern. The absorptions for silicon nitrides and hydrides such as SiNN, Si(NN)_2_, SiH_2_, and SiH_4_ have been reported previously and agree with our assignments (Supplementary Figs. 7, 8)^32,38,39^.

**Table 1:** Observed and calculated (CCSD(T)/aug-cc-pVDZ) infrared absorptions (cm^−1^) and H/D ratios for products of the reactions of Si atoms with H_2_ molecules in solid N_2_

	H_2_		D_2_		H/D ratio		Mode assignment
	Calc.	Obs.	Calc.	Obs.	Calc.	Obs.	
**A** H_2_Si(*μ*-N)_2_SiH_2_ (^1^A_1_, C_2v_)							
^14^N	2275.3 (150)	covered	1642.0 (76)	1586.1	1.3857	/	H_2_Si-SiH_2_ stretch, b_2_
	2230.9 (163)	2165.3	1601.7 (69)	1551.3	1.3928	1.3958	H_2_Si-SiH_2_ stretch, a_1_
	964.4 (308)	937.8	689.5 (79)	679.2	1.3988	1.3807	2SiH_2_ bend, b_2_
	874.3 (170)	857.7	872.6 (288)	855.3	1.0019	1.0028	Si(NN)Si ring, b_2_
^15^N	2275.2 (137)	covered	1642.0 (75)	1586.1	1.3856	/	H_2_Si-SiH_2_ stretch, b_2_
	2230.8 (161)	2165.3	1601.7 (68)	1551.3	1.3928	1.3958	H_2_Si-SiH_2_ stretch, a_1_
	964.4 (287)	937.2	688.5 (75)	679.2	1.4008	1.3799	2SiH_2_ bend, b_2_
	855.7 (180)	covered	854.9 (282)	834.7	1.0009	/	Si(NN)Si ring, b_2_
**B** H_2_SiNN(H_2_) (^1^A_1_, C_s_)							
^14^N	3733.3 (86)	3569.3	2641.2 (43)	2530.6	1.4135	1.4105	H-H stretch, a'
	2034.4 (215)	1991.6	1464.3 (110)	1425.9	1.3894	1.3967	SiH_2_ stretch, a”
	2024.8 (143)	1983.6	1454.4 (76)	1421.8	1.3922	1.3951	SiH_2_ stretch, a'
	1129.3 (50)	1097.5	803.8 (26)	804.4	1.4050	1.3644	H-H twist, a'
	982.2 (78)	952.4	704.5 (39)	684.4	1.3942	1.3916	SiH_2_ scissor, a'
^15^N	3733.3 (86)	3569.3	2641.2 (43)	2530.6	1.4135	1.4105	H-H stretch, a'
	2034.3 (215)	1991.6	1464.3 (111)	1425.9	1.3893	1.3967	SiH_2_ stretch, a”
	2024.7 (143)	1983.6	1454.4 (76)	1421.8	1.3921	1.3951	SiH_2_ stretch, a'
	1129.2 (50)	1097.5	803.8 (26)	804.4	1.4050	1.3644	H-H twist, a'
	982.2 (78)	952.4	704.5 (39)	684.4	1.3941	1.3916	SiH_2_ scissor, a'
**C** HNSiNH (^1^A_1_, C_2_)							
^14^N	3559.9 (176)	3564.8	2607.4 (124)	2662.5	1.3653	1.3389	NH stretch, υ_as_
	1228.1 (106)	1197.6	1194.2 (128)	1164.8	1.0284	1.0282	NSiN stretch, υ_as_
^15^N	3551.8 (132)	3556.2	2595.2 (120)	2647.3	1.3686	1.3433	NH stretch, υ_as_
	1211.9 (37)	1175.9	1177.8 (123)	1143.2	1.0290	1.0286	NSiN stretch, υ_as_

The absorptions of the cyclic species **A** were observed upon λ > 220 nm irradiation and increased markedly upon the second λ > 220 nm irradiation (Fig. 2). The symmetric and antisymmetric D-Si-D modes were observed at 1551.3 and 1586.1 cm^–1^, respectively (Fig. 3b and Supplementary Fig. 1). Only the symmetric H-Si-H mode at 2165.3 cm^−1^ can be detected, leading to a H/D ratio of 1.3958. This observation is most likely due to a strong SiH_4_ band that covers the antisymmetric mode. In addition, the strongest absorption at 937.8 cm^−1^ of the H-Si-H bending mode shifted to 679.2 cm^−1^ in the deuterium experiment (H/D isotopic ratio 1.3807) (Fig. 3b and Supplementary Fig. 1), and in the ^15^N_2_ experiment a slight shift of 0.6 to 937.2 cm^−1^ was observed (Fig. 3d and Supplementary Fig. 4). Furthermore, a band for species **A** at 855.3 cm^−1^ in the D_2_/^14^N_2_ experiment (Fig. 3b) shifted by 20.6 to 834.7 cm^−1^ in the D_2_/^15^N_2_ experiment (Fig. 3f and Supplementary Fig. 6), which results in a ^14^N/^15^N ratio of 1.0247. The band can be assigned to the Si-N-Si stretching mode. The corresponding H_2_/^14^N_2_ experiment shows a band at 857.7 cm^–1^ (Fig. 3a). Its counterpart in the H_2_/^15^N_2_ experiment was covered by a broad signal of Si_2_H_6_ centered at 835.4 cm^−1^ (Fig. 3d and Supplementary Fig. 4).

When the isotopologues HD or a mixture of 5% H_2_ and 5% D_2_ as a hydrogen source were used, band distributions were observed for the bands of species **A** (Fig. 3c and Supplementary Figs. 2, 3). There are four possible isomers of **A** (**A**_**3**_**, A**_**4**_**, A**_**5**_, and **A**_**6**_) relating to 2H and 2D (see Supplementary Tables 1, 2). The 15% HD/^14^N_2_ experiment shows bands at 2167.4 and 2162.4 cm^–1^ (Si-H symmetric vibration), 1580.1, 1575.8, 1556.8, and 1550.5 cm^–1^ (Si-D vibration), 847.5 and 845.3 cm^–1^ (SiHD bend) which correspond to the isomers **A**_**4**_ (2167.4, 1575.8, and 847.5 cm^–1^), **A**_**5**_ (1556.8 and 845.3 cm^–1^) and **A**_**6**_ (1580.1, 1550.5, and 845.3 cm^–1^). The experiments using a mixture of 5% H_2_/5% D_2_ in ^14^N_2_ show three main isomers **A**_**1**_, **A**_**2**_, **A**_**3**_ with four groups of double bands at 2165.3 and 2163.0 cm^–1^ (Si-H vibration), 1586.1, 1577.2, 1551.3, and 1549.2 cm^–1^ (Si-D vibration), 946.4 and 937.8 cm^–1^ (SiH_2_ and SiD_2_ bend), 857.7 and 855.3 cm^–1^(Si(NN)Si ring); among these the bands at 2163.0, 1577.2, 1549.2, and 946.4 cm^–1^ can be attributed to **A**_**3**_. All bands are listed in Table 1 and Supplementary Tables 1–3. The identification of molecule **A** is also based on the excellent agreement of the observed and calculated frequencies at the CCSD(T) and B3LYP level of theory.

**Fig. 4:**
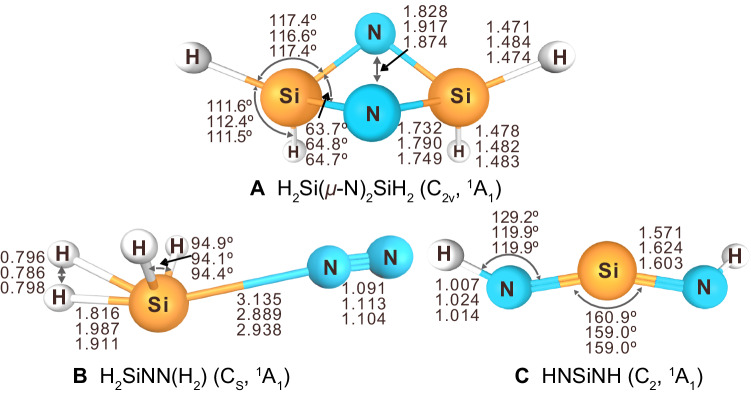
Calculated geometries of A, B, and C. H_2_Si(*μ*-N)_2_SiH_2_ (**A**), H_2_SiNN(H_2_) (**B**), HNSiNH (**C**) based on B3LYP/6-311 + + G(3*df*,3*pd*) (upper), CCSD(T)/aug-cc-pVDZ (middle), and CCSD(T)/aug-cc-pVTZ (lower) methods (bond lengths in Å and bond angles in degree).

The calculated N–N distance in **A** is 1.828 Å at the B3LYP level (Fig. 4), which is significantly longer than the N–N single bond of diphenylhydrazine [d(N-N): 1.394 Å]^40^. A Mayer bond order of the N–N bond for molecule **A** is 0.689 computed at the B3LYP/6-311 G(3*df*,3*pd*) level. This suggests that the N≡N triple bond is cleaved by the two SiH_2_ units. The resonance structures of compound **A** have been provided by NBO-based Natural Resonance Theory (NRT) analysis (Supplementary Fig. 9)^41–43^. The ring inversion barrier of the puckered ring system of **A** was computed to be 6.2 kcal mol^–1^ at the B3LYP/6-311 + + G (3df, 3pd) level (Supplementary Fig. 10). As known from other cyclic main-group species, aromaticity is an important factor to stabilize both the transition state and the product during the N_2_ fixation process. To evaluate the compound’s aromaticity, computational chemistry is an effective tool^44,45^, and the aromaticity of the four-membered ring in **A** has been confirmed by the multi-center bond order (MCBO) indexes, gauge including magnetically induced current (GIMIC)^46,47^, the nucleus-independent chemical shift (NICS)^48^ and the electron density of delocalized bonds (EDDB)^49,50^ analyses. MCBO index, which is also known as the multi-center index (MCI) to evaluate aromaticity from the aspect of electron delocalization properties^51–53^. The MCBO index of **A** is 0.3046, similar to that of FB(*μ*-N)_2_BF (0.3190), which also has an aromatic four-membered B_2_N_2_ ring^16^. GIMIC method was calculated at B3LYP/6-311 + +g (3*df*, 3*pd*) level and a net diamagnetic ring current of 11.4 nA T^–1^ in **A** similar to the typical aromatic molecule benzene (11.8 nA T^–1^)^54^ could demonstrate the aromaticity of complex **A** (Supplementary Fig. 11). What’s more, the isotropic magnetic shielding tensor has been the most popular index for measuring aromaticity^55^, and the average NICS(1)_av_ index can serve as a probe of aromaticity in nonplanar molecular systems. (Supplementary Fig. 12)^56^. The large negative NICS(1)_av_ and NICS(0) indexes of –29.0 and –39.1 obtained at the center of gravity of species **A** indicate its aromatic character. Canonical molecular orbital natural chemical shielding (CMO-NICS(1)_ZZ_)^57^ was calculated at B3LYP/ 6-311 + + G(3*df*,3*pd*) level to separate the σ and π contributions of canonical molecular orbital, and larger diatropic contribution of –14.6 ppm from σ orbitals compared with −11.9 ppm from π orbitals (Supplementary Fig. 13) indicates σ aromaticity dominated in **A**, consistent with the EDDB analysis where 1.55 electrons delocalization was calculated (Supplementary Table 8) which are comparable to the number of delocalized electrons in L_2_Si_2_P_2_ (L = PhC(NtBu)_2_, 1.77 electrons)^58^ and the tetraatomic boron specie (1.57 electrons)^59^ and σ aromaticity is also the dominant one (60%).

H_2_SiNN(H_2_) (**B***)* shows absorptions at 3569.3, 1991.6, 1983.6, 1097.5, and 952.4 cm^−1^ which appeared already upon codeposition (Fig. 2) and markedly increased upon λ > 300 nm irradiation with the obvious reduction of the SiNN. It decreased by 60% upon λ > 220 nm irradiation. The strong bands at 1991.6, 1983.6, and 952.4 cm^−1^ are assigned to the Si-H stretching and bending modes of the SiH_2_ subunit, which shifted to 1425.9, 1421.8, 684.4 cm^−1^ in the deuterium experiment (Fig. 3b and Supplementary Fig. 1), giving isotopic frequency ratios of 1.3967, 1.3951, and 1.3916, respectively. Moreover, two weak bands located at 3569.3 and 1097.5 cm^−1^ have been observed with H_2_, which shift to 2530.6 and 804.4 cm^−1^ with D_2_, exhibiting a H/D ratio of 1.4105 and 1.3644. They can be assigned to an H-H stretching and bending mode (Supplementary Fig. 1). These bands appear at 3110.3, 3102.4, and 977.0 cm^−1^ in the HD sample (Supplementary Fig. 2). Some bands at 1987.2, 1984.5, 1425.3, and 830.0 cm^−1^ turn up in 5% H_2_/5% D_2_ or HD experiments (Fig. 3c and Supplementary Figs. 2, 3) and are assigned to a SiHD stretching and bending mode. All these results suggest that **B** contains one side-on coordinated H_2_ molecule. The computed frequencies of H_2_SiNN(H_2_) are provided in Table 1 and Supplementary Table 4 and show a very good agreement between theory and experiment. Furthermore, in low H_2_ concentrations (less than 2%), sharp bands at 2274.2, 2013.6, 2009.4, and 928.8 cm^−1^ were observed, which have been assigned to H_2_SiN_2_ (Supplementary Figs. 14, 15)^36^. As shown in Supplementary Fig. 16, with an increase of H_2_, these doublet bands disappear, and the absorptions of species **B** will strongly be enhanced. This suggests an additional H_2_ coordination to H_2_SiN_2_ and, therefore, built species **B**. The ν(Si-H) stretching for **B** at 1991.6 and 1983.6 cm^–1^ is lower than these modes for H_2_SiN_2_ and the ν(N-N) stretching is higher, which is in good agreement with the B3LYP and CCSD(T) calculations (Supplementary Tables 9, 10). We performed Tesla coil discharge experiments to confirm the assignment of compound **B** (Supplementary Figs. 17–19). The absorptions of compound **B** increased after annealing to 33 K at the expense of H_2_SiN_2_ (Supplementary Fig. 17) and became even more intense with the increase of H_2_ concentrations ranging from 0 to 10% (Supplementary Fig. 18). This proves that H_2_ plays an important role in coordinating to H_2_SiN_2_ to give species **B**. Compound **B** might be described as a pseudo SiH_4_ forming a very weak adduct with dinitrogen with a bond distance slightly shorter than the van der Waals distance of 3.7 Å and the N-N stretching frequency is too weak to be observed.

Two additional bands at 3564.8 and 1197.6 cm^−1^ assigned to a new molecule HNSiNH (**C**) have been observed by codeposition. They increased on both λ > 300 nm and λ > 220 nm irradiation at the expense of the absorptions of H_2_SiN_2_ and SiNN (see Fig. 2 and Supplementary Tables 5, 11). These bands belong to the N–H and N–Si–N stretching modes of the species HNSiNH. Additional isotope experiments were performed using a mixture of D_2_/N_2_. They show absorptions of the corresponding isotopologues at 2662.5 cm^−1^ (H/D isotopic ratio 1.3389), which is very close to the isotopic ratio of HNSi in a 4 K argon matrix (H/D isotopic ratio 1.3424)^60^, and at 1164.8 cm^−1^ (H/D isotopic ratio 1.0282), see Fig. 3b and Supplementary Fig. 1. With H_2_/^15^N_2_, the counterpart bands were observed at 3556.2 cm^–1^ (^14^N/^15^N isotopic ratio 1.0024) and 1175.9 cm^−1^ (^14^N/^15^N isotopic ratio 1.0186) also shown in Fig. 3d and Supplementary Fig. 4. With D_2_/^15^N_2_, the two bands shifted to 2647.3 and 1143.2 cm^−1^ (Fig. 3f and Supplementary Fig. 6). Moreover, in our HD experiment, a band at 1178.4 cm^−1^ reveals an isotopic triplet indicating the involvement of two equivalent hydrogen atoms (Fig. 3c and Supplementary Figs. 2, 3). The Si–N bond length of **C**, calculated to be 1.571 Å at the B3LYP level (Fig. 4), is much shorter than the Si–N single bond (1.87 Å) and is close to the N=Si bond in HN=Si (1.559 Å)^61–64^ and the N=Si monomer with a double bond of 1.572 Å^65,66^.

**Fig. 5:**
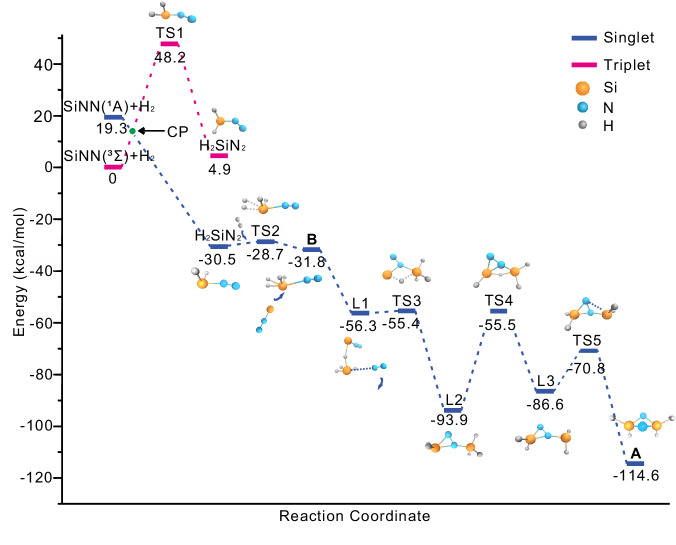
Potential energy surface for the reaction of *B* + SiNN → *A* + N_2_ computed at the B3LYP/6-311 + + G(3*df*, 3*pd*) level of theory. The unit of relative energy is kcal/mol.

As shown in Fig. 5, SiNN (^3^Σ) was supposed to be a starting compound to give the complex H_2_SiNN (^1^A_1_) with H_2_ upon λ > 300 nm irradiation. In the next step, H_2_SiNN reacts with a second H_2_ molecule to form complex **B**. The corresponding barrier was computed to be only 1.8 kcal/mol at the DFT level, which is in accordance with the increase of H_2_SiNN and complex **B** and the decrease of SiNN upon λ > 300 nm irradiation. Although H_2_Si: could not be observed directly in the spectrum, it can still be assumed that H_2_Si: is formed either in the reaction of silicon with hydrogen or via SiH_x_ decomposition, and in a dinitrogen atmosphere will further react with N_2_ or N_2_ and H_2_ to form H_2_SiNN and H_2_SiNN(H_2_). Further reaction of H_2_SiNN(H_2_) with SiNN forms the H_2_Si(*μ*-N)_2_SiH_2_ (**A**) complex through hydrogen transfer, forming two SiH_2_ subunits and finally leading to an NN triple bond cleavage (Fig. 5 and Supplementary Figs. 20–22). In principle, the cryogenic conditions of the matrix provide a good environment in that both H_2_SiNN(H_2_) and SiNN synergistically react as Lewis bases and initiate a step-wise reduction of the N_2_ moiety. In the Tesla coil discharge reactions of SiH_4_ with or without H_2_ in excess solid N_2_, both H_2_SiN_2_ and species **B** can be observed, while species **A** is missing due to the lack of SiNN (Supplementary Fig. 23g–i). In the reactions of laser-ablated Si atoms with 10% SiH_4_ in solid nitrogen, species **A** is also not generated because of the absence of species **B**. This shows that both **B** and SiNN are essential for the formation of product **A** (Supplementary Fig. 23d–f).

**Fig. 6:**
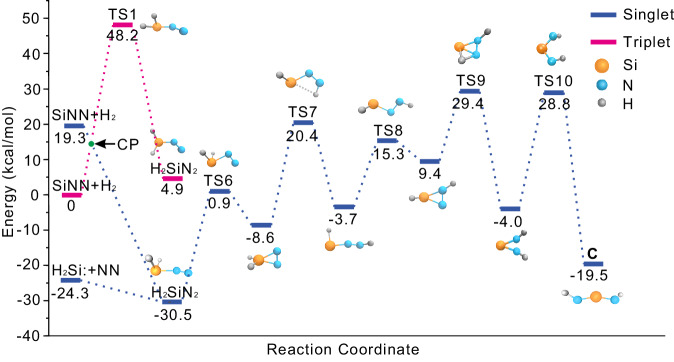
Potential energy surface for the reaction of H_2_Si + N_2_ → *C* and SiNN + H_2_ → *C* computed at the B3LYP/6-311 + + G(3*df*, 3*pd*) level of theory. The unit of relative energy is kcal/mol.

Based on quantum-chemical calculations, the nitrogen atoms in **A** show a more negative partial charge of –1.07 e, while the Si atoms carry a more positive partial charge of 1.45 e compared to compound **B** (Supplementary Table 12). As shown at the energy surface (Fig. 5), A is computed to be exothermic by 114.6 kcal mol^–1^ with the highest activation barrier of 38.4 kcal mol^–1^. Note that the activation of CO to react to the ethynediolate dianion [OCCO]^2–^ has been achieved by a bis-silylene^31^. In addition, SiNN (^1^A_1_, *C*_2v_) also leads to the formation of **C**, with an activation barrier of 32.8 kcal mol^–1^. **C** is observed in freshly deposited samples, and it increased markedly after λ > 220 nm irradiation, in which the laser-ablated energy provided in the codeposition process and the irradiation energy at λ > 220 nm most likely support the formation separately. SiNN (^1^A_1_, *C*_2v_) reacts with H_2_ to form H_2_SiN_2_ and then isomerizes to ***C*** through H transfer and N-N bond cleavage (Fig. 6 and Supplementary Figs. 24, 25). Similar exothermic reactions could occur when H-substituted groups, such as CH_3_ and Ph, are applied (Supplementary Fig. 26). Similar to **NHC1-H**^67^, H_2_Si displays the smallest singlet–triplet energy gap and lowest ΔE to give H_2_SiNN, showing the great potential for dinitrogen activation.

**Fig. 7:**
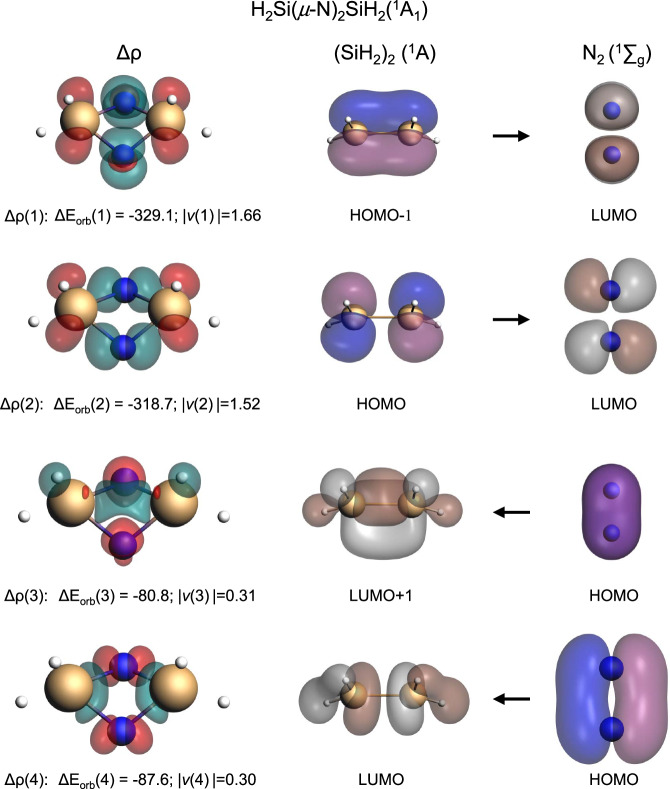
Shape of the deformation densities, Δ*ρ*_(1)-(4)_ of H_2_Si(*μ*-N)_2_SiH_2_ corresponding to ΔE_orb(1)_-ΔE_orb(4)_ and the associated fragment orbitals at the meta-Hybrid/M06-2X/TZP level. Isosurface values are 0.004 a.u. The eigenvalues $\left|{{{{{{\rm{v}}}}}}}_{{{{{{\rm{n}}}}}}}\right|$ give the size of the charge migration in e. The direction of the charge flow of the deformation densities is red→green.

The EDA-NOCV method was used to elucidate the peculiar stability of the bonding nature in **A***.* As shown in Fig. 7 the neutral fragments N_2_ (^1^Ʃ_g_) and (SiH_2_)_2_ (^1^A) in the singlet state, which refer to the symmetry-allowed dissociation products, have been selected as interacting moieties that address the question about all changes along the bond formation between two neutral fragments^68^. The numerical results are shown in Supplementary Table 13 and the breakdown of the orbital interaction into pairwise orbital interactions reveals that the dominant orbital stabilization, Δ*E*_orb(1)_ (–329.1 kcal mol^–1^) and Δ*E*_orb(2)_ (–318.7 kcal mol^–1^) comes from the back-donation of the HOMO-1 (mainly 3*p*_x_ of the Si atom) and the HOMO (mainly 3*p*_x_ of the Si atom) of the (SiH_2_)_2_ moiety into the two perpendicular π* MOs of the N_2_ ligands, known as push effect. The most interesting orbital interactions are Δ*E*_orb(3)_ and Δ*E*_orb(4)_, which contribute to the donation of π MO electrons of the N_2_ ligands to the LUMO + 1 (3*p*_z_ of Si atom and 1 *s* of H atom) and LUMO (mainly 3*s*, 3*p*_y_, 3*p*_z_, and 4*s* of Si atom) of the (SiH_2_)_2_ fragment (pull effect). This is unlike the end-on complex H_2_SiNN^42,43^ with a σ-donation and a π back-donation, in which the σ-donation only plays a bonding role for the Si-N bond. However, for complex **A**, H_2_Si**:** shows both interactions, which donates electron density to π*-antibonding orbitals of N_2_ and removes electron density from the π-bonding orbitals of N_2_ and, therefore, cooperatively cleaves the NN triple bond^69^. In addition, our EDA-NOCV results are illustrated in Supplementary Table 14 for **A** using neutral and charged fragments as interacting moieties. The smallest Δ*E*_orb_ values are found when the doubly charged species (SiH_2_)_2_^2+^ (^3^A) and (N_2_)^2-^ (^3^∑_g_) are used for the calculations, which is a measure for the best description of the bonds finally formed^70,71^. The orbital term Δ*E*_orb_ accounts for 70% of the total attraction between the neutral units. However, the dominance of covalent bonding disappears when the final bonding situation is analyzed. The electrostatic part of the attractive interactions constitutes greater than 50% of the total attraction. The shape of the deformation densities, Δ*ρ*_(1)-(4)_ of H_2_Si(*μ*-N)_2_SiH_2_ using (SiH_2_)_2_^2−^ and N_2_^2−^ as interacting fragments were shown in Supplementary Fig. 27. When the bond finally formed, (SiH_2_)_2_^2+^ (^3^B_2_) and (N_2_)^2−^ (^3^∑_g_) still basically follow the rules of “push and pull”, but the donation from N_2_^2−^ is stronger.

In this work, three activated dinitrogen species, H_2_Si(*μ*-N)_2_SiH_2_, H_2_SiNN(H_2_), and HNSiNH, have been identified by isotopic substitution experiments under cryogenic conditions in matrix-isolation experiments in conjunction with quantum-chemical calculations. The N≡N triple bond was activated and broken by the synergistic interaction of two SiH_2_ moieties in low-temperature matrix circumstances to form the stable aromatic ring system Si_2_N_2_. The SiNN is supposed to be an important starting compound to form the complex H_2_SiNN(H_2_) (B) with H_2_, which then further reacts with SiNN to build the final product H_2_Si(*μ*-N)_2_SiH_2_ (A). The EDA-NOCV calculations support a dual interaction of H_2_Si, which is able to donate electron density to π*-antibonding orbitals of N_2_ and remove electron density from the π-bonding orbitals, leading to the cleavage of the N_2_ triple bond and the formation of H_2_Si(*μ*-N)_2_SiH_2_. This research work might open a different way to functionalize and activate dinitrogen molecules.

## Methods

### Matrix-isolation experiments

A Nd:YAG laser fundamental(1064 nm, 10 Hz repetition rate with 10 ns pulse width and 20−50 mJ/pulse) was focused on a rotating silicon target (Alfa Aesar), generating a bright plume. The laser-ablated silicon atoms reacting with H_2_, D_2_, HD, and H_2_ + D_2_ mixtures in solid N_2_ and ^15^N_2_, were condensed at 4 K using a closed-cycle helium refrigerator (Sumitomo Heavy Industries Model SRDK-408D2). Infrared spectra were recorded on a Bruker 80 v spectrometer at 0.5 cm^−1^ resolution between 4000 and 400 cm^−1^ using a HgCdTe range B detector. Further experimental details are provided in the Supplementary Information.

### Quantum chemistry calculation

All of the structures were optimized at Post-HF (CCSD(T)) and complementary density functional theory (DFT) methods and the vibration frequencies were computed analytically via Gaussian 09 program^72^. The bonding nature in **A** was investigated using energy decomposition analysis (EDA) combined with the natural orbitals for the chemical valence (NOCV) method. The analyses for the aromaticity were calculated with the Gaussian 09 program, the Multiwfn code^53^, NBO 6.0 program^57^, GIMIC2.0 program^47,73^, and the RunEDDB program^49,50^, respectively. Further quantum-chemical details are provided in the Supplementary Information.

### Supplementary information

Supplementary Information

Peer Review File

## Data Availability

All data generated in this study are provided in the Article and Supplementary Information. The experiment data that support the findings of this study are available from the corresponding author upon request.
