# Author Correction: N_2_ cleavage by silylene and formation of H_2_Si(*μ*-N)_2_SiH_2_

**DOI:** 10.1038/s41467-024-48898-7

**Published:** 2024-05-24

**Authors:** Liyan Cai, Bing Xu, Juanjuan Cheng, Fei Cong, Sebastian Riedel, Xuefeng Wang

**Affiliations:** 1 School of Chemical Science and Engineering, Shanghai Key Lab of Chemical Assessment and Sustainability, Tongji University, Shanghai, 200092 China; 2 Institut für Chemie und Biochemie – Anorganische Chemie, Freie Universität Berlin, Fabeckstrasse 34-36, D-14195 Berlin, Germany

**Keywords:** Reaction mechanisms, Structure elucidation, Chemical bonding

Correction to: *Nature Communications* 10.1038/s41467-024-48064-z, published online 08 May 2024

In this article the graphics relating to Figs 5, 6, and 7 had been interchanged; the figure(s) should have appeared as shown below.

Figure 5:
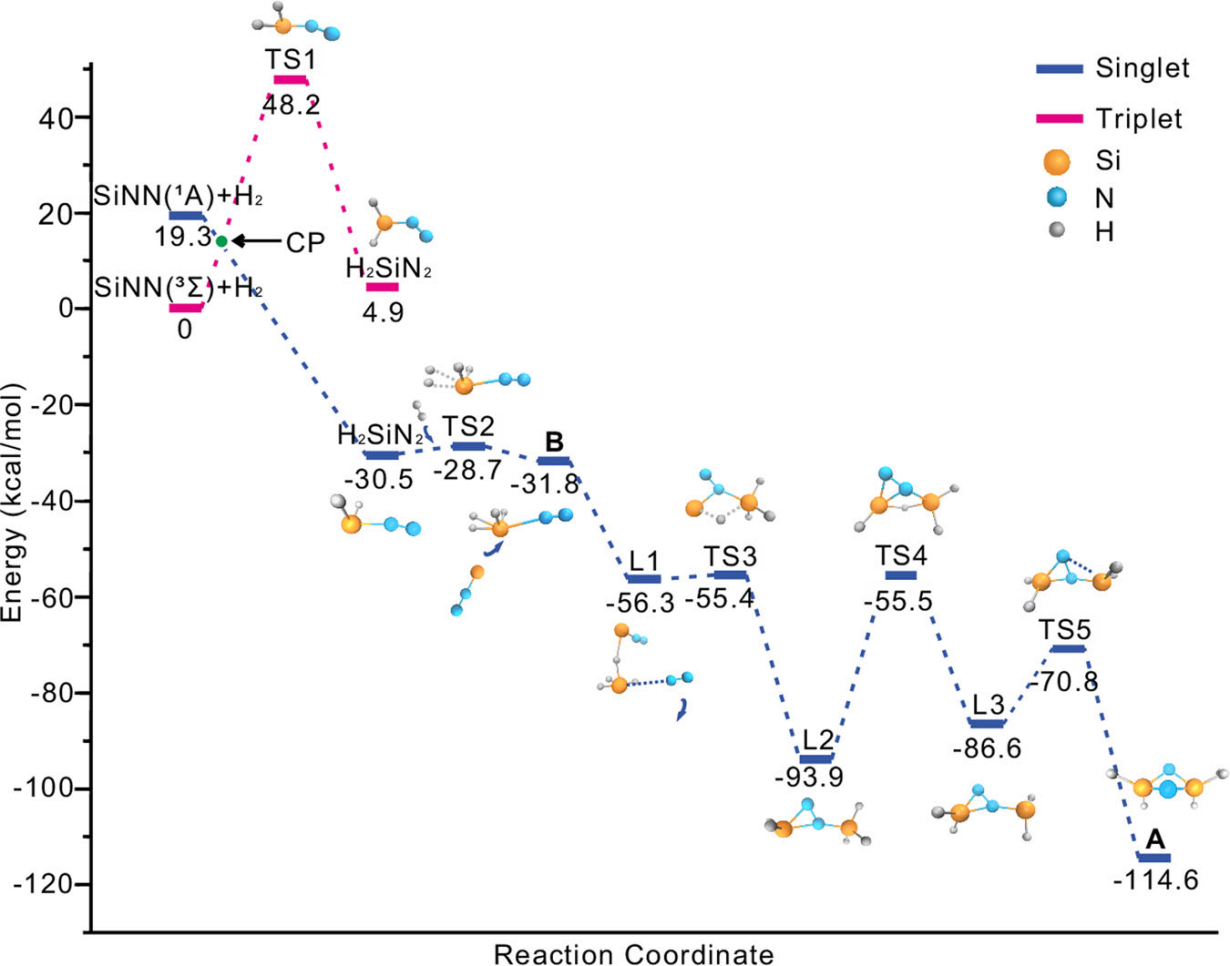


Figure 6:
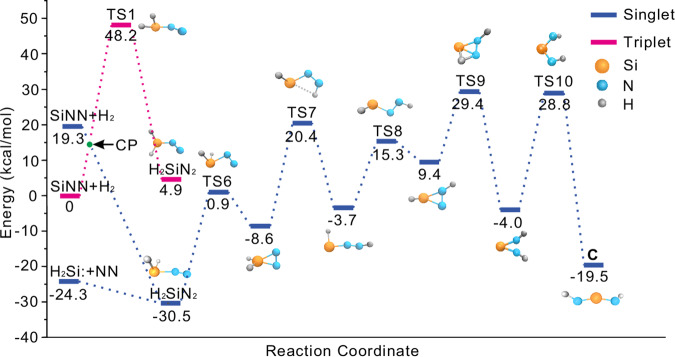


Figure 7:
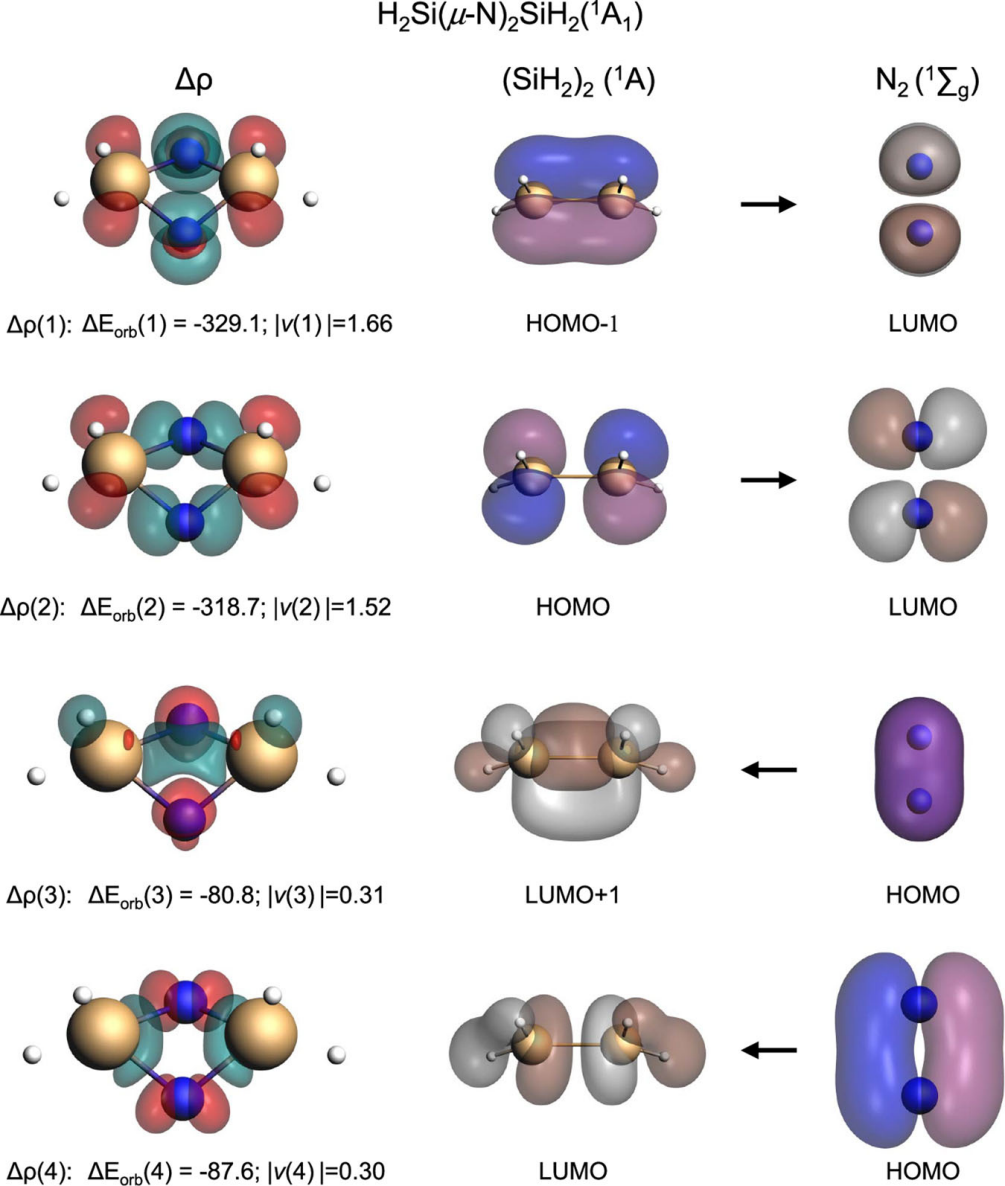


The original article has been corrected.

