# Supplementary material for: N2 cleavage by silylene and formation of H2Si(μ-N)2SiH2
Source: Nat Commun. 2024 May 8;15:3848. doi: 10.1038/s41467-024-48064-z (PMC11078988; doi:10.1038/s41467-024-48064-z)
Supplement: Supplementary file 1 — Supplementary Information [file 41467_2024_48064_MOESM1_ESM.pdf]

Supplementary Information

N_2 Cleavage by Silylene and Formation of $H_2Si(\mu\text{-}N)_2SiH_2$

Liyan Cai[1], Bing Xu[1]*, Juanjuan Cheng[1], Fei Cong[1], Sebastian Riedel[2]* and Xuefeng Wang[1]*

1 Table of Contents

Supplementary Figures Page 1

Supplementary Figure 1. Infrared spectra of the laser-ablated Si atoms reactions with 10% D_2 in $^{14}N_2$ matrix at 4 K. Page 1

Supplementary Figure 2. Infrared spectra of the laser-ablated Si atoms reactions with 15% HD in $^{14}N_2$ matrix at 4 K. Page 1

Supplementary Figure 3. Infrared spectra of the laser-ablated Si atoms reactions with Si + 5% H_2 + 5% D_2 in $^{14}N_2$ matrix at 4 K. Page 2

Supplementary Figure 4. Infrared spectra of the laser-ablated Si atoms reactions with Si + 10% H_2 in $^{15}N_2$ matrix at 4 K. Page 2

Supplementary Figure 5. Infrared spectra of the laser-ablated Si atoms reactions with Si + 10% H_2 in $^{14}N_2$ + $^{15}N_2$ matrix at 4 K. Page 3

Supplementary Figure 6**.** Infrared spectra of the laser-ablated Si atoms reactions with Si + 10% D_2 in $^{15}N_2$ matrix at 4 K. Page 3

Supplementary Figure 7**.** Infrared spectra of the laser-ablated Si atoms reactions with pure $^{14}N_2$ at 4 K. Page 4

Supplementary Figure 8. Infrared spectra of the laser-ablated Si atoms reactions with 5% N_2 + 5% H_2 in Ar at 4 K. Page 4

Supplementary Figure 9. Natural Resonance Theory (NRT) description of product $H_2Si(\mu\text{-}N)_2SiH_2$ (5 candidate reference structure). Page 5

Supplementary Figure 10. Transition state for the ring inversion of **A** ($^1A_1$, D_{2h}) and its frequencies (cm^{-1}). Page 5

Supplementary Figure 11. The iso-surface(left) and direction(right) of the ring-current density in $H_2Si(\mu\text{-}N)_2SiH_2$(**A**). Page 5

Supplementary Figure 12. Nucleus-independent chemical shift (NICS) values for nonplanar cyclic-SiNSiN. Page 6

Supplementary Figure 13. CMO-NICS(1)$_{ZZ}$ of $H_2Si(\mu\text{-}N)_2SiH_2$ (**A**). Page 6

Supplementary Figure 14. Infrared spectra in the 2040-1980 cm^{-1} region for the laser-ablated Si atoms reactions with hydrogen in excess solid nitrogen at 4 K. Page 7

Supplementary Figure 15. Infrared spectra of the laser-ablated Si atoms reactions with low concentrations of H_2, D_2 and HD in excess solid $^{14}N_2$ ($^{15}N_2$) at 4 K after codeposition for 120 min and the second λ > 220 nm irradiation for 10 min. Page 7

Supplementary Figure 16. Infrared spectra of the reaction products for the laser-ablated Si atoms reactions with hydrogen in excess solid nitrogen at 4 K. Page 8

Supplementary Figure 17. Infrared spectra of the Tesla coil discharge of SiH_4 reactions with different concentrations of H_2 in excess solid N_2 at 4 K after codeposition for 120 min, λ > 300 nm irradiation for 10 min, annealing to 25K and annealing to 33K. Page 9

Supplementary Figure 18. Infrared spectra of the Tesla coil discharge of 10% SiH_4 reactions with different concentrations of H_2 in excess solid N_2 at 4 K after annealing to 33K. Page 9

Supplementary Figure 19. Infrared spectra of the Tesla coil discharge of 10% SiH4 reactions with 10% H2 in excess solid $^{14}N_2$ at 4 K. Page 10

Supplementary Figure 20. Transition state structure (**TS1-TS5**) ($^1A_1$, C_1) and its frequencies. Page 10

Supplementary Figure 21. Potential Energy Surface of H_2SiN_2 from H_2 and SiNN on the H-H bond length. Page 11

Supplementary Figure 22. Potential Energy Surface of L1 from **B** and SiNN on the H-Si bond length. Page 11

Supplementary Figure 23. Infrared spectra of different experiments to produce H_2SiN_2 and **B** in excess solid nitrogen at 4 K. Page 12

Supplementary Figure 24. Transition state structure (**TS6-TS10**) ($^1A_1$, C_1) and its frequencies. Page 13

Supplementary Figure 25. Potential Energy Surface of H_2SiN_2 from H_2Si and NN on the Si-NN bond length from 1.955 to 4.955Å at the B3LYP/6-311++G(3df, 3pd) level of theory. Page 13

Supplementary Figure 26. Structures and zero-point energies of R_2Si:, R_2SiNN and $R_2Si(\mu\text{-}N)_2SiR_2$ (R=F, Cl, Br, CN, CF_3, Ph, H and CH_3). Page 14

Supplementary Figure 27. Using $(SiH_2)_2^{2-}$ and N_2^{2-} as interacting fragment and the shape of the deformation densities, $\Delta\rho_{(1)\text{-}(4)}$ of $H_2Si(\mu\text{-}N)_2SiH_2$ corresponding to $\Delta E_{orb(1)}$-$\Delta E_{orb(4)}$ and the associated fragment orbitals at the meta-Hybrid/M06-2X/TZP leve. Page 15

Supplementary Figure 28. Laplacian distribution of the charge density for $H_2Si(\mu\text{-}N)_2SiH_2$. Page 15

Supplementary Figure 29. Isosurface map of electron localization function (ELF) for $H_2Si(\mu\text{-}N)_2SiH_2$. Page 16

Supplementary Tables Page 17

Supplementary Table 1. Infrared absorptions (cm^{-1}) observed for products of the reactions of Si atoms with H_2 molecules in solid N_2. Page 17

Supplementary Table 2. Observed and Calculated Fundamental Frequencies (cm^{-1}) of $H_2Si(\mu\text{-}N)_2SiH_2$ (C_{2v}, $^1A_1$) in ^{14}N matrix. Page 18

Supplementary Table 3. Observed and Calculated Fundamental Frequencies (cm^{-1}) of $H_2Si(\mu\text{-}^{15}N)_2SiH_2$ (C_{2v}, $^1A_1$) in ^{15}N matrix. Page 21

Supplementary Table 4. Observed and Calculated Fundamental Frequencies (cm^{-1}) of $H_2SiNN(H_2)$ (C_S, $^1A_1$). Page 22

Supplementary Table 5. Observed and Calculated Fundamental Frequencies (cm^{-1}) of HNSiNH (C_2, $^1A_1$). Page 25

Supplementary Table 6. Comparison between the observed and calculated vibrational frequencies (cm^{-1}) and isotopic frequency ratios of the new products. Page 26

Supplementary Table 7. Infrared absorptions (cm^{-1}) observed for SiNN, NNSiNN, and SiH_2 in solid $^{14}N_2/^{15}N_2$. Page 26

Supplementary Table 8. Electron Density of Delocalized Bonds (EDDB) of $H_2Si(\mu\text{-}N)_2SiH_2$ (**A**). Page 26

Supplementary Table 9. Observed and calculated infrared absorptions (cm^{-1}) for products of **B** and H_2SiN_2. Page 27

Supplementary Table 10. Observed and Calculated Fundamental Frequencies (cm^{-1}) of H_2SiN_2 in low H_2 concentration. Page 28

Supplementary Table 11. Observed and Calculated Fundamental Frequencies (cm^{-1}) of $HNSiNH(NN)_n$, n=0-2. Page 29

Supplementary Table 12. NBO reaults for $H_2Si(\mu\text{-}N)_2SiH_2$, $H_2SiNN(H_2)$, H_2SiNN and HNSiNH. Page 30

Supplementary Table 13. EDA-NOCV results of $H_2Si(\mu\text{-}N)_2SiH_2$ at the meta-Hybrid/M06-2X/TZP level taking $(SiH_2)_2$ and N_2 in the singlet states as interacting fragments. Page 31

Supplementary Table 14. EDA-NOCV results of $H_2Si(\mu\text{-}N)_2SiH_2$ at the meta-Hybrid/M06-2X/TZP level taking $(SiH_2)_2$ and N_2 in the different charged states as interacting fragments. Page 31

Supplementary Methods Page 32

Supplementary Notes Page 33

Supplementary References Page 33

Total Number of Pages-35

Supplementary Figures

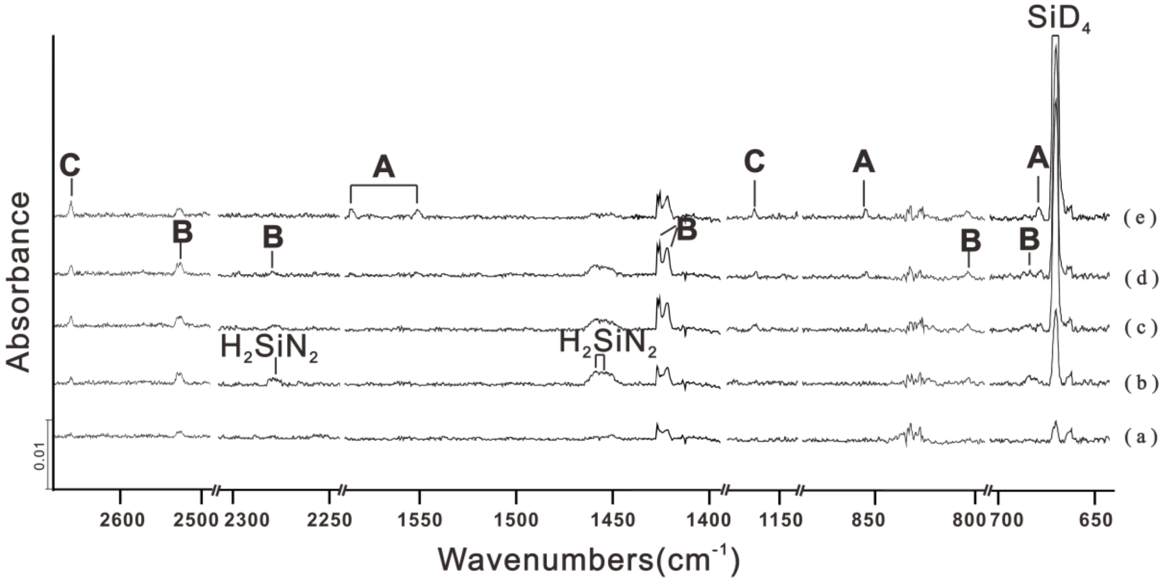

Supplementary Figure 1. Infrared spectra of the laser-ablated Si atoms reactions with 10% D_2 in $^{14}N_2$ matrix at 4 K. (a) codeposition of Si + 10% D_2 in $^{14}N_2$ matrix for 120 min; (b) after λ > 300 nm irradiation for 10 min; (c) after λ > 220 nm irradiation for 10 min; (d) after annealing to 7 K; (e) after λ > 220 nm irradiation for 10 min. **A**: $H_2Si(\mu\text{-}N)_2SiH_2$, **B**: $H_2SiNN(H_2)$, **C**: HNSiNH

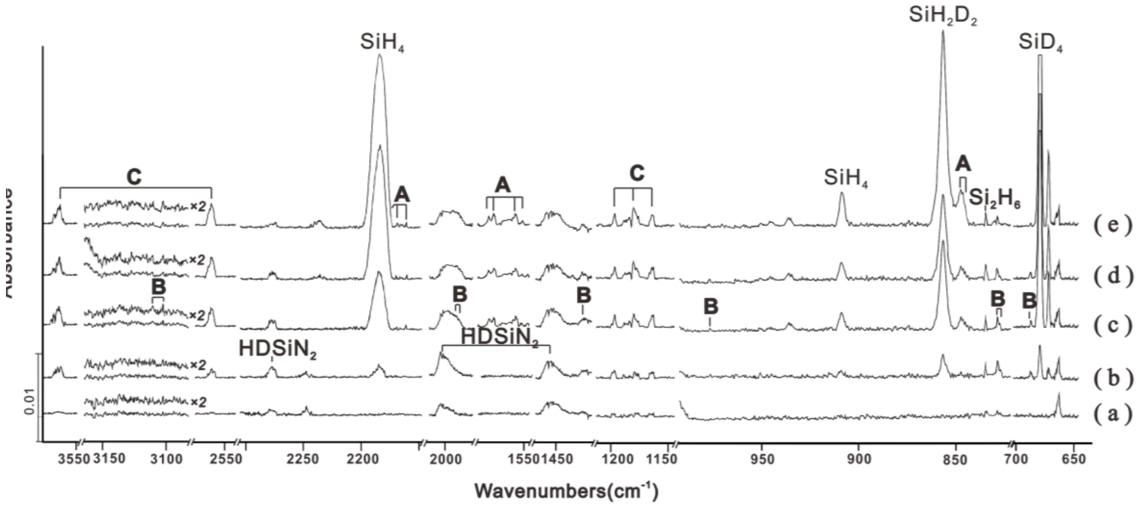

Supplementary Figure 2. Infrared spectra of the laser-ablated Si atoms reactions with 15% HD in $^{14}N_2$ matrix at 4 K. (a) codeposition of Si + 15% HD in $^{14}N_2$ matrix for 120 min; (b) after λ > 300 nm irradiation for 10 min; (c) after λ > 220 nm irradiation for 10 min; (d) after annealing to 7 K; (e) after λ > 220 nm irradiation for 10 min. **A**: $H_2Si(\mu\text{-}N)_2SiH_2$, **B**: $H_2SiNN(H_2)$, **C**: HNSiNH

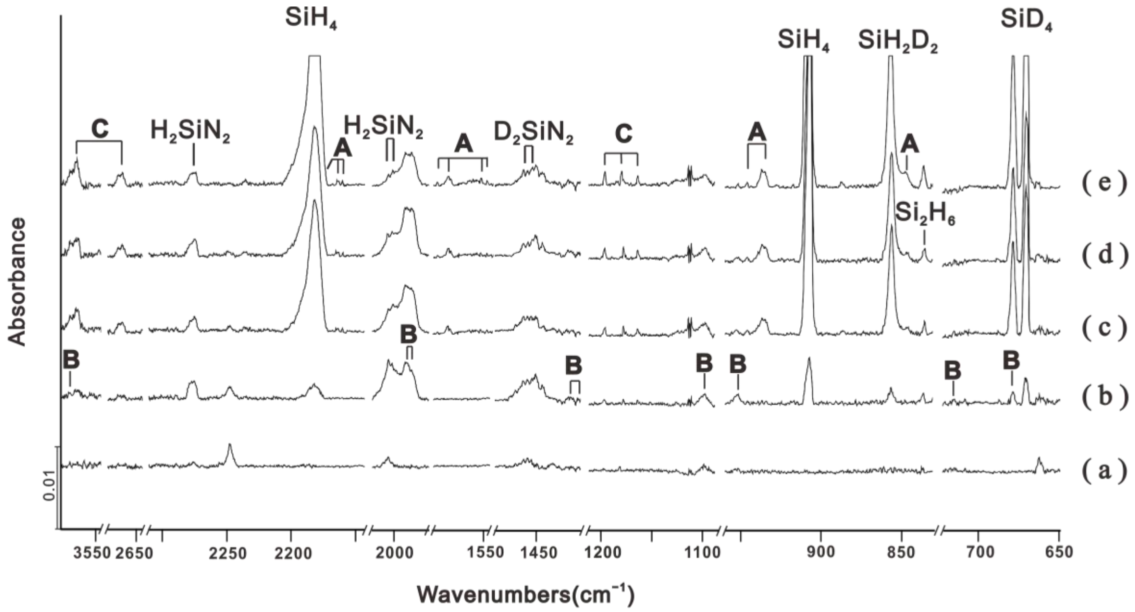

Supplementary Figure 3. Infrared spectra of the laser-ablated Si atoms reactions with Si + 5% H_2 + 5% D_2 in $^{14}N_2$ matrix at 4 K. (a) codeposition of Si + 5% H_2 + 5% D_2 in $^{14}N_2$ matrix for 120 min; (b) after λ > 300 nm irradiation for 10 min; (c) after λ > 220 nm irradiation for 10 min; (d) after annealing to 7 K; (e) after λ > 220 nm irradiation for 10 min. **A**: $H_2Si(\mu\text{-}N)_2SiH_2$, **B**: $H_2SiNN(H_2)$, **C**: HNSiNH

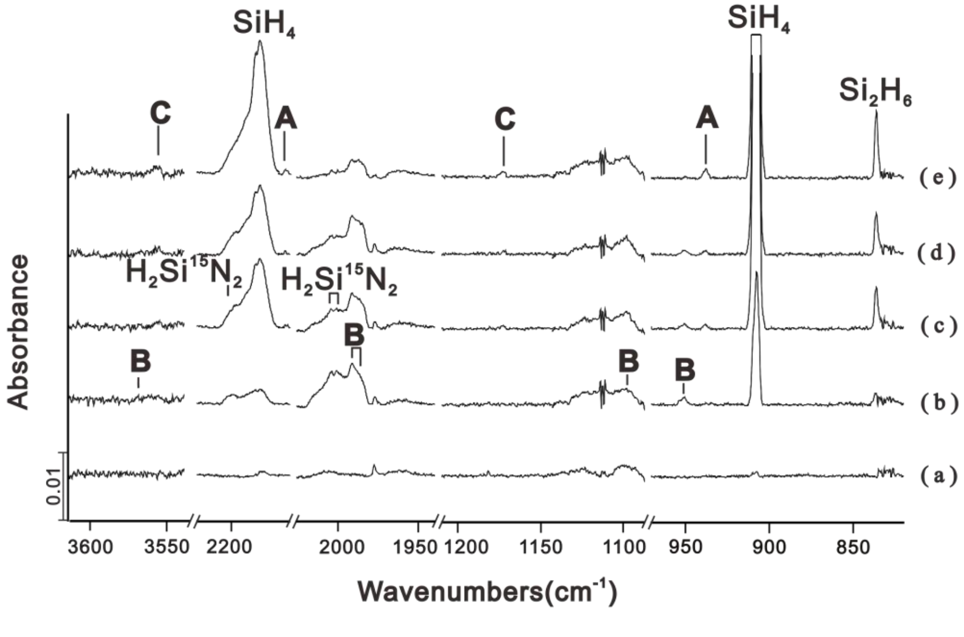

Supplementary Figure 4. Infrared spectra of the laser-ablated Si atoms reactions with Si + 10% H_2 in $^{15}N_2$ matrix at 4 K. (a) codeposition of Si + 10% H_2 in $^{15}N_2$ matrix for 120 min; (b) after λ > 300 nm irradiation for 10 min; (c) after λ > 220 nm irradiation for 10 min; (d) after annealing to 7 K; (e) after λ > 220 nm irradiation for 10 min. **A**: $H_2Si(\mu\text{-}N)_2SiH_2$, **B**: $H_2SiNN(H_2)$, **C**: HNSiNH

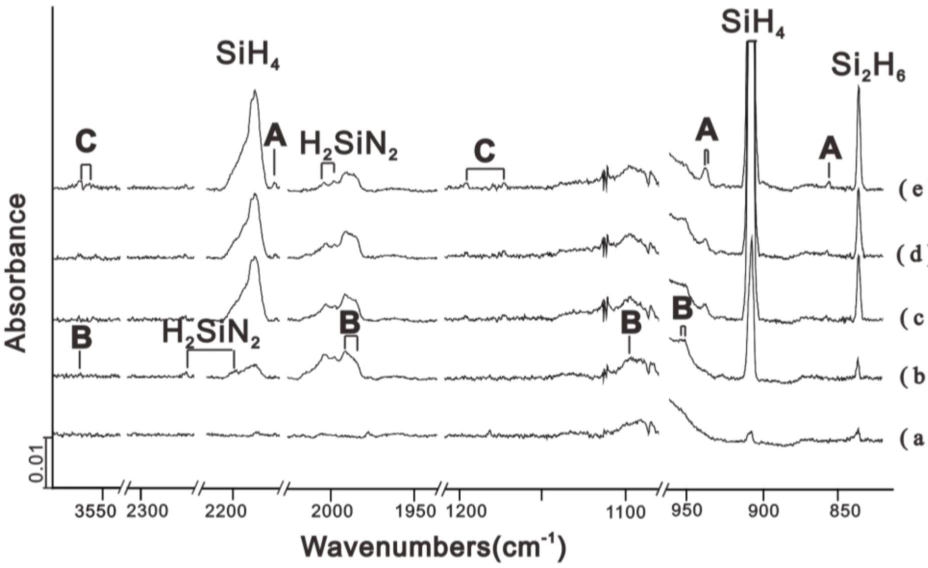

Supplementary Figure 5. Infrared spectra of the laser-ablated Si atoms reactions with Si + 10% H_2 in $^{14}N_2$ + $^{15}N_2$ matrix at 4 K. (a) codeposition of Si + 10% H_2 in $^{14}N_2$ + $^{15}N_2$ matrix for 120 min; (b) after λ > 300 nm irradiation for 10 min; (c) after λ > 220 nm irradiation for 10 min; (d) after annealing to 7 K; (e) after λ > 220 nm irradiation for 10 min. **A**: $H_2Si(\mu\text{-}N)_2SiH_2$, **B**: $H_2SiNN(H_2)$, **C**: HNSiNH

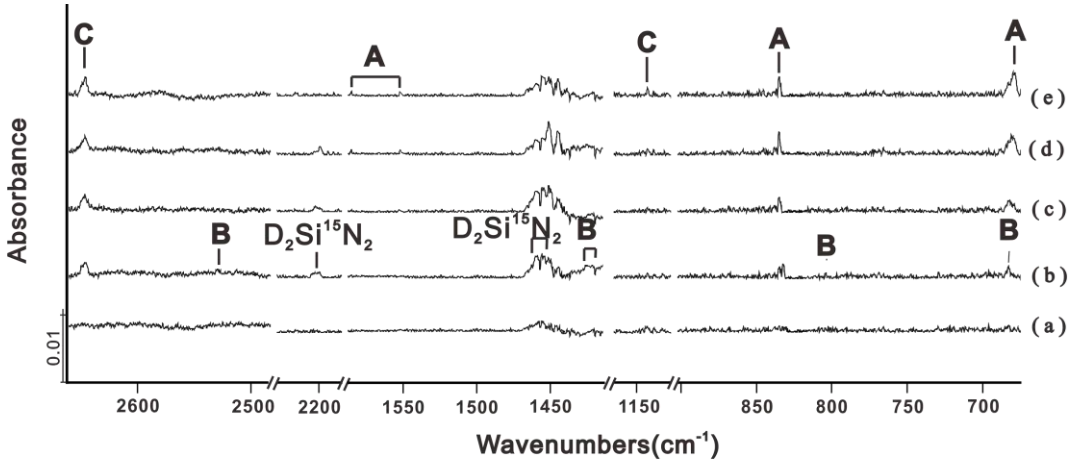

Supplementary Figure 6. Infrared spectra of the laser-ablated Si atoms reactions with Si + 10% D_2 in $^{15}N_2$ matrix at 4 K. (a) codeposition of Si + 10% D_2 in $^{15}N_2$ matrix for 120 min; (b) after λ > 300 nm irradiation for 10 min; (c) after λ > 220 nm irradiation for 10 min; (d) after annealing to 7 K; (e) after λ > 220 nm irradiation for 10 min. **A**: $H_2Si(\mu\text{-}N)_2SiH_2$, **B**: $H_2SiNN(H_2)$, **C**: HNSiNH

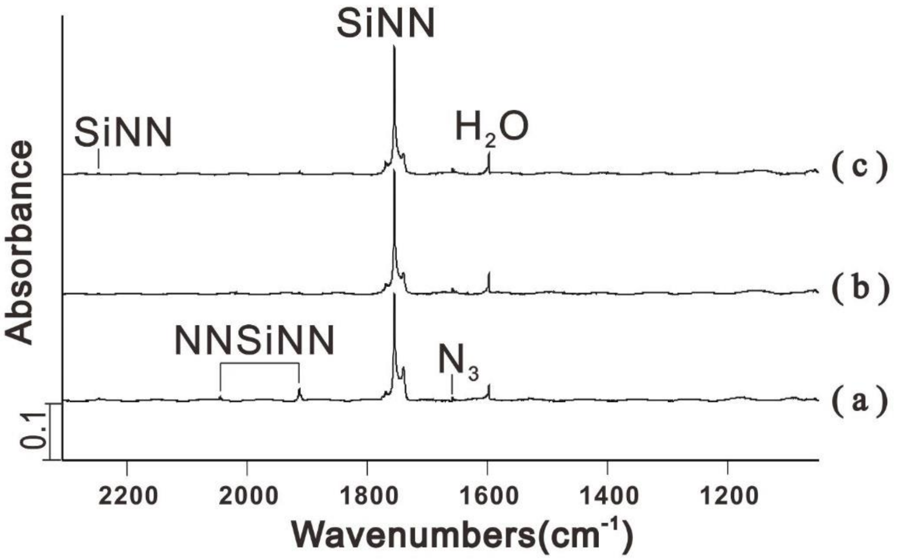

Supplementary Figure 7 Infrared spectra of the laser-ablated Si atoms reactions with pure $^{14}N_2$ at 4 K. (a) codeposition of Si + $^{14}N_2$ for 120 min; (b) after λ > 300 nm irradiation for 10 min; (c) after λ > 220 nm irradiation for 10 min.

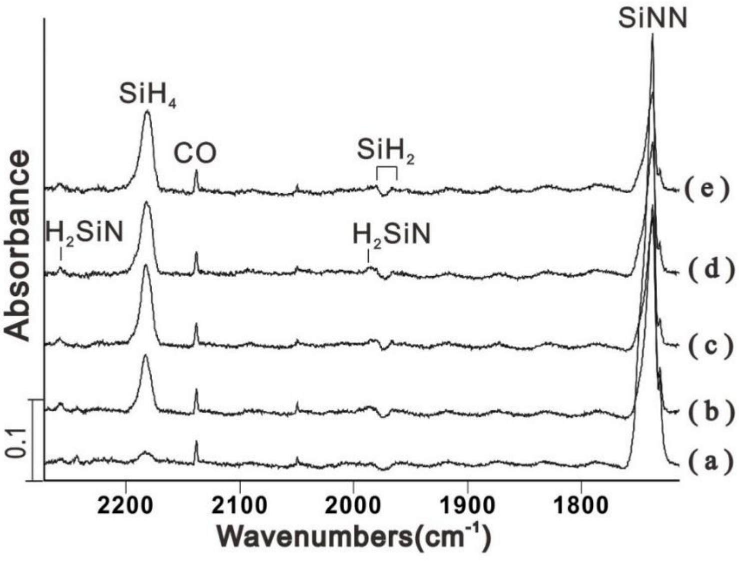

Supplementary Figure 8 Infrared spectra of the laser-ablated Si atoms reactions with 5% N_2 + 5% H_2 in Ar at 4 K. (a) codeposition of Si + $^{14}N_2$ for 120 min; (b) after λ > 300 nm irradiation for 10 min; (c) after λ > 220 nm irradiation for 10 min; (d) after annealing to 15K; (e) after λ > 220 nm irradiation for 10 min.

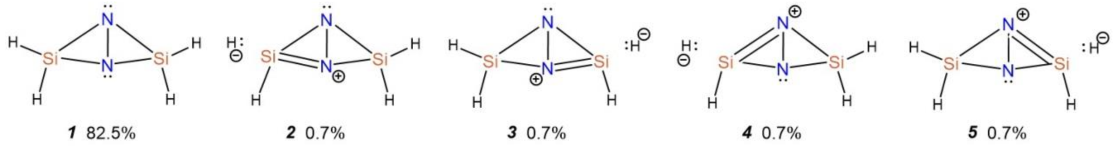

Supplementary Figure 9. Natural Resonance Theory (NRT) description of product $H_2Si(\mu$-N$)_2SiH_2$ (5 candidate reference structure).

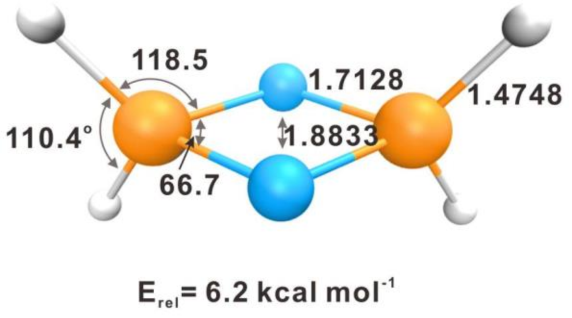

Frequency	Infrared
-197.5	26
472.9	0
481.4	0
504.9	0
564.2	23
670.8	132
707.8	0
716.8	0
821.7	0
848.1	120
907.7	0
1010.1	151
1046.8	0
1051.5	567
2248.4	245
2252.3	0
2253.8	263
2256.1	0

Supplementary Figure 10. Transition state for the ring inversion of **A** ($^1A_1$, D_{2h}) and its frequencies (cm^{-1}) obtained at the B3LYP/6-311++G(3df, 3pd) level of theory. The relative energy is 6.2 kcal mol^{-1} with respect to the puckered ring.

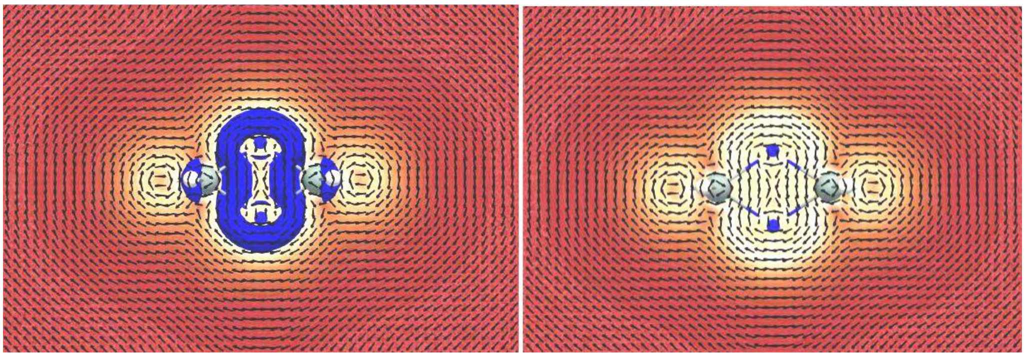

Supplementary Figure 11. The iso-surface(left) and direction(right) of the ring-current density in $H_2Si(\mu$-N$)_2SiH_2$ calculated at the B3LYP/6-311++g (3df, 3pd) level. Diatropic contribution is indicated with blue.

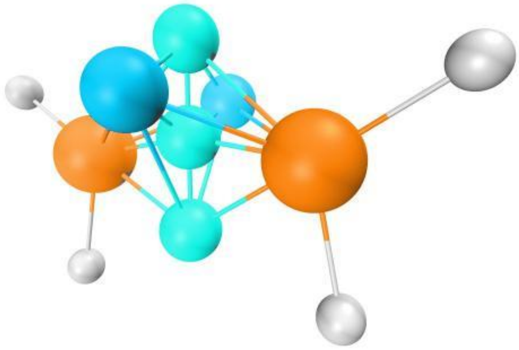

Supplementary Figure 12. Nucleus-independent chemical shift (NICS) values for nonplanar cyclic-SiNSiN in the mass center of the molecule (NICS(0)), 1Å above (NICS(1)) and 1Å (NICS(-1)) below the ring center , calculated at the B3LYP/6-311++G(3df, 3pd) level of theory.

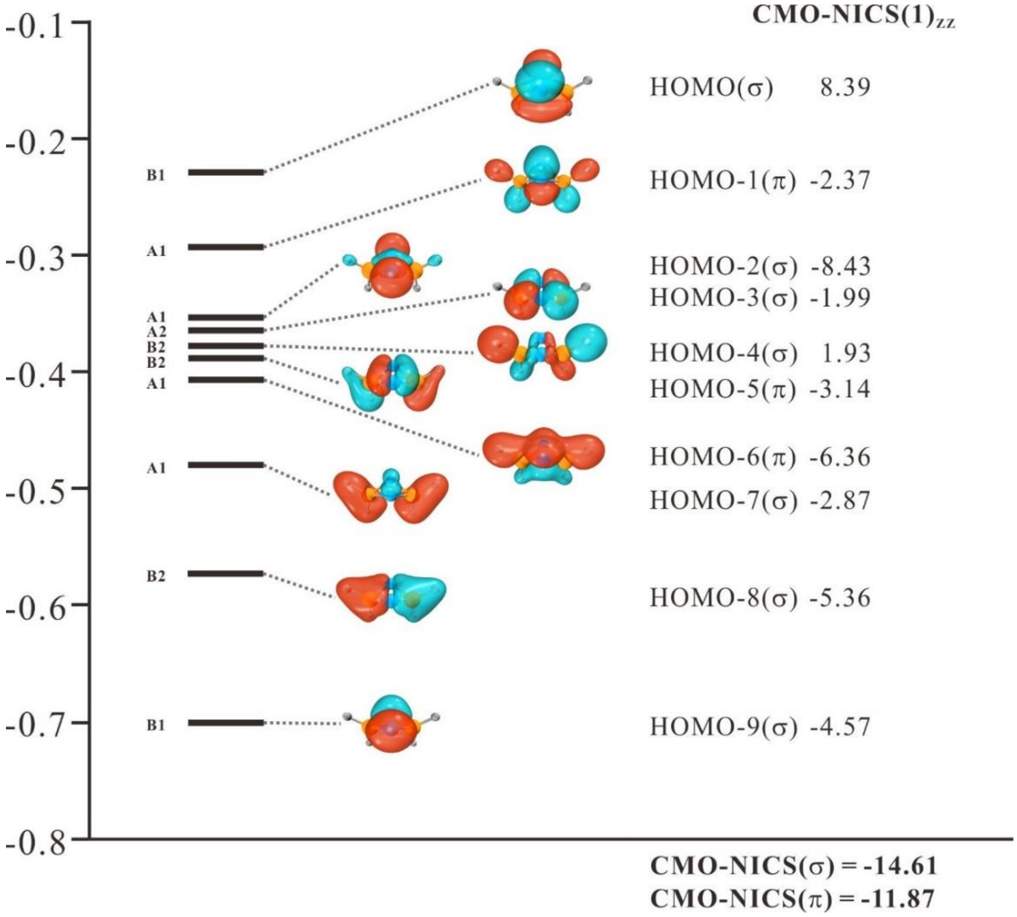

Supplementary Figure 13. CMO-NICS(1)$_{ZZ}$ of $H_2Si(\mu\text{-}N)_2SiH_2$ at B3LYP/6-311++G(3df,3pd) level. NICS values are in ppm. MO energies in a. u. are given on the left side.

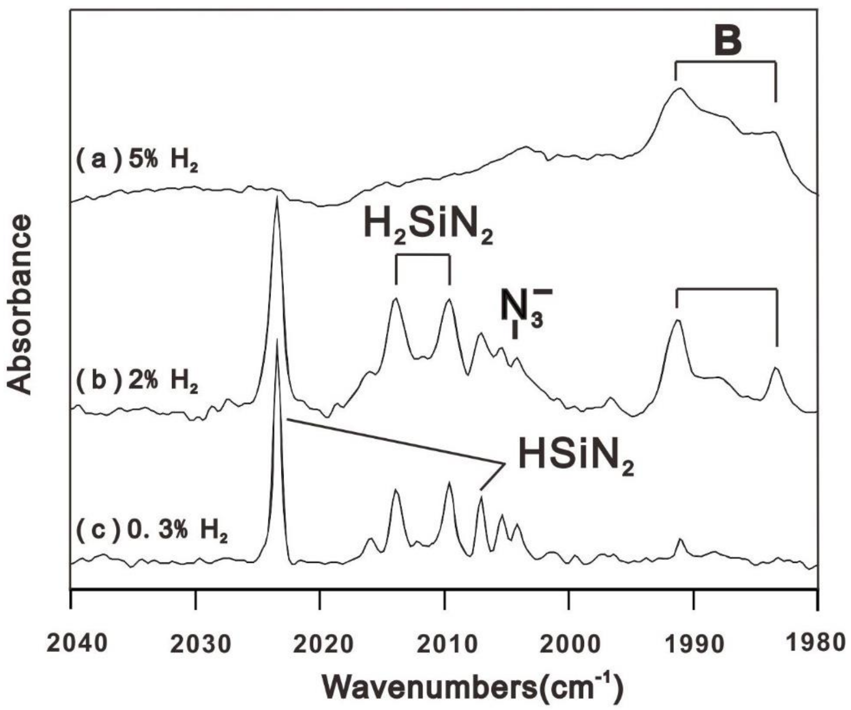

Supplementary Figure 14. Infrared spectra in the 2040-1980 cm^{-1} region for the laser-ablated Si atoms reactions with hydrogen in excess solid nitrogen at 4 K. (spectra taken after 10 min of >220 nm irradiation): (a) Si +5 % H_2, (b) Si + 2% H_2; (c) Si + 0.3% H_2; **B**: $H_2SiNN(H_2)$.

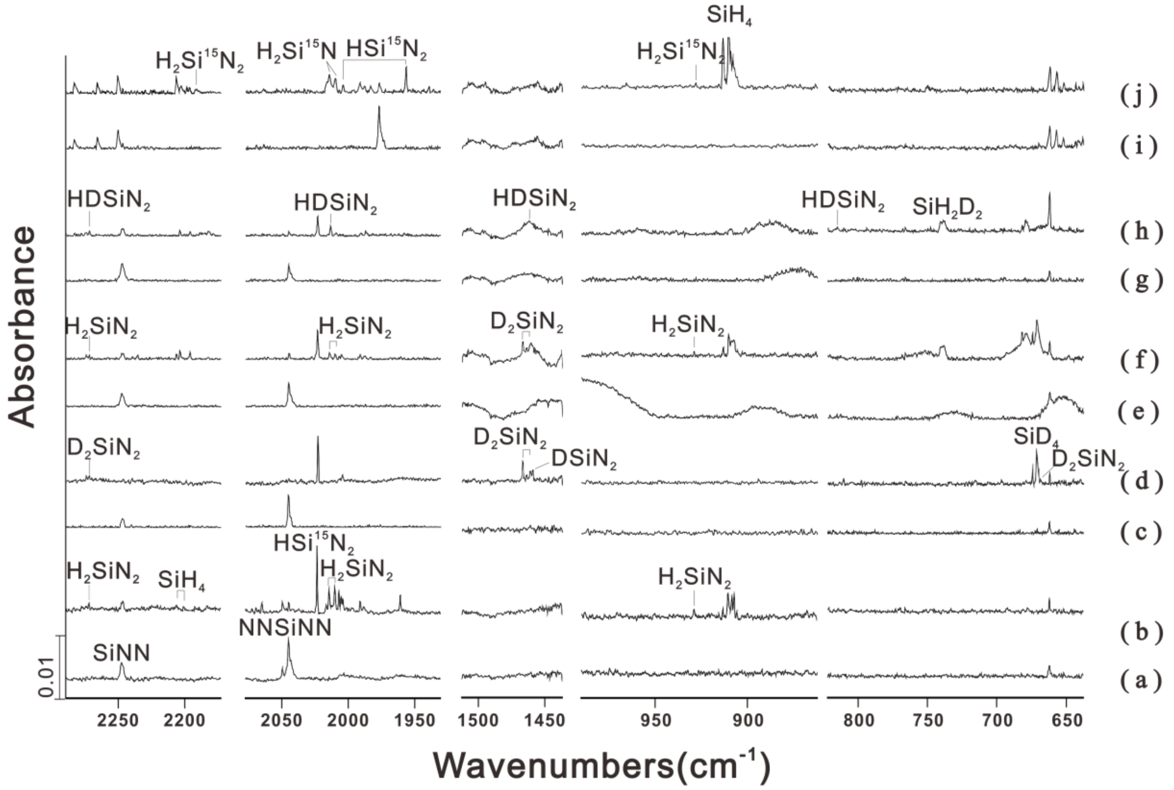

Supplementary Figure 15. Infrared spectra of the laser-ablated Si atoms reactions with low concentrations of H_2, D_2 and HD in excess solid $^{14}N_2$ ($^{15}N_2$) at 4 K after codeposition for 120

min and the second λ > 220 nm irradiation for 10 min. (a and b) Si + 0.3% $H_2/^{14}N^{14}N$; (c and d) Si + 1% $D_2/^{14}N^{14}N$; (e and f) Si + 1% H_2 + 1% $D_2/^{14}N^{14}N$; (g and h) Si + 1% $HD/^{15}N^{15}N$; (i and j) Si + 1% $H_2/^{15}N^{15}N$.

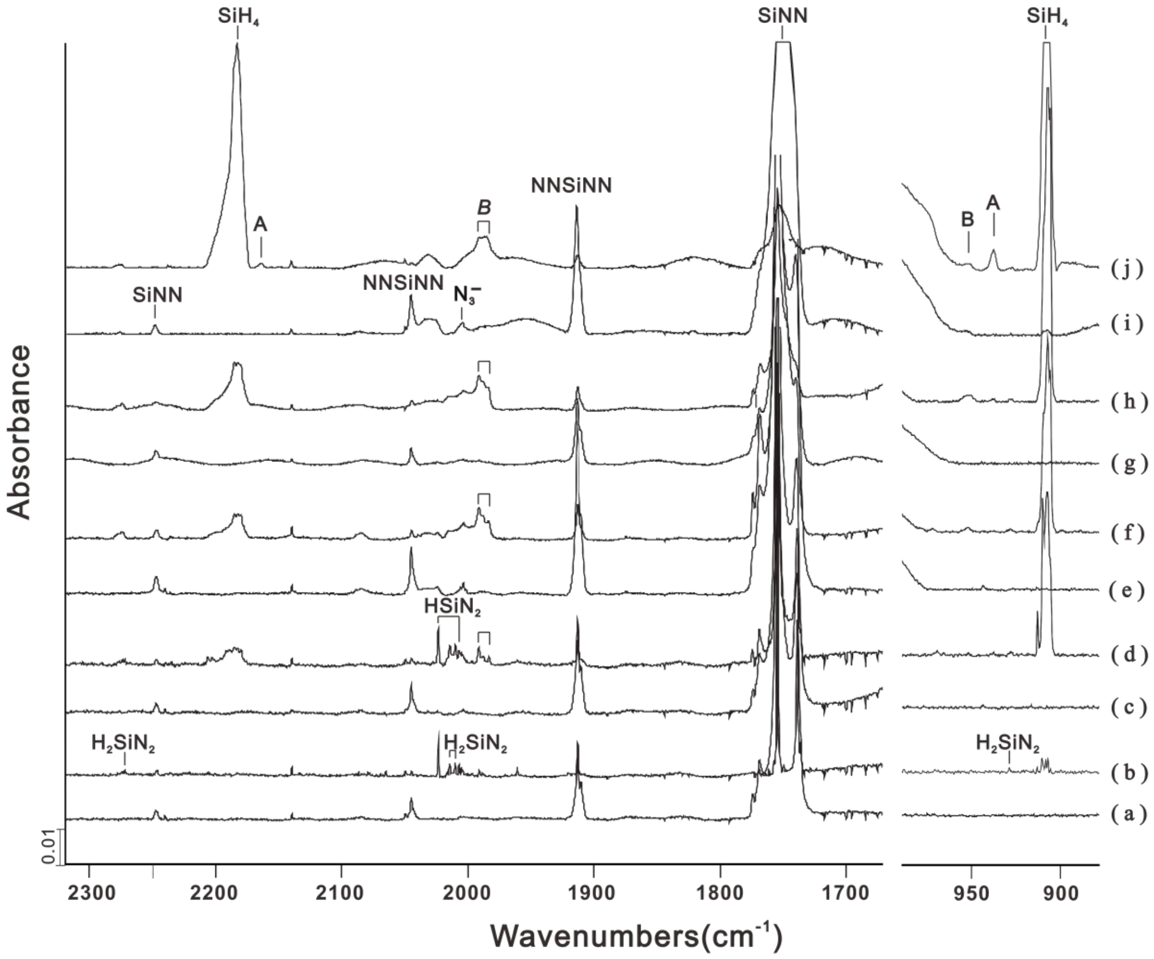

Supplementary Figure 16. Infrared spectra of the reaction products for the laser-ablated Si atoms reactions with hydrogen in excess solid nitrogen at 4 K. (spectra taken after codeposition and 10 min of >220 nm irradiation, respectively): (a and b) Si + 0.3% H_2, (c and d) Si + 2% H_2; (e and f) Si + 3% H_2; (g and h) Si + 5% H_2; (i and j) Si + 10% H_2. **A**: $H_2Si(\mu\text{-}N)_2SiH_2$, **B**: $H_2SiNN(H_2)$

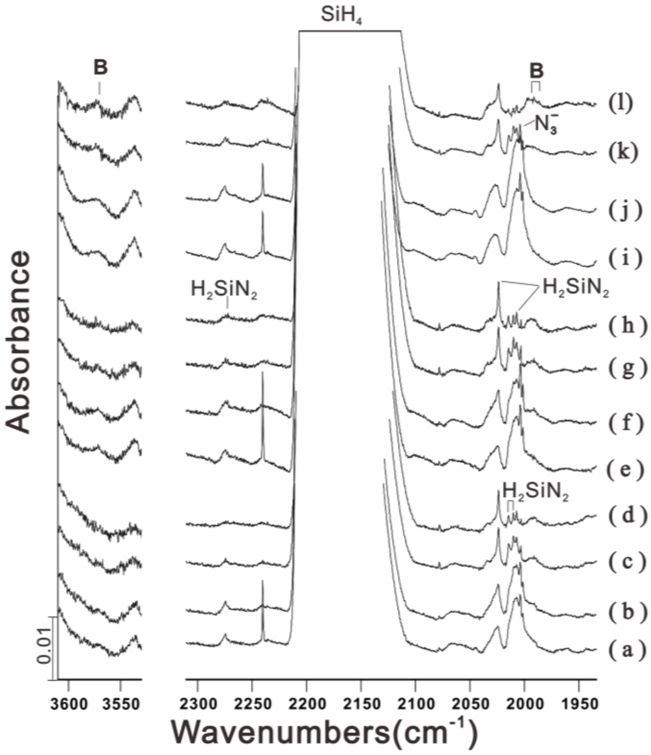

Supplementary Figure 17. Infrared spectra of the Tesla coil discharge of 10% SiH_4 reactions with different concentrations of H_2 in excess solid $^{14}N_2$ at 4 K after codeposition for 120 min, λ > 300 nm irradiation for 10 min, annealing to 25K and annealing to 33K. (a - d) 10% SiH_4/NN; (e - h) 10% SiH_4 + 5% H_2/NN; (i - l) 10% SiH_4 + 10% H_2/NN. **B**: $H_2SiNN(H_2)$

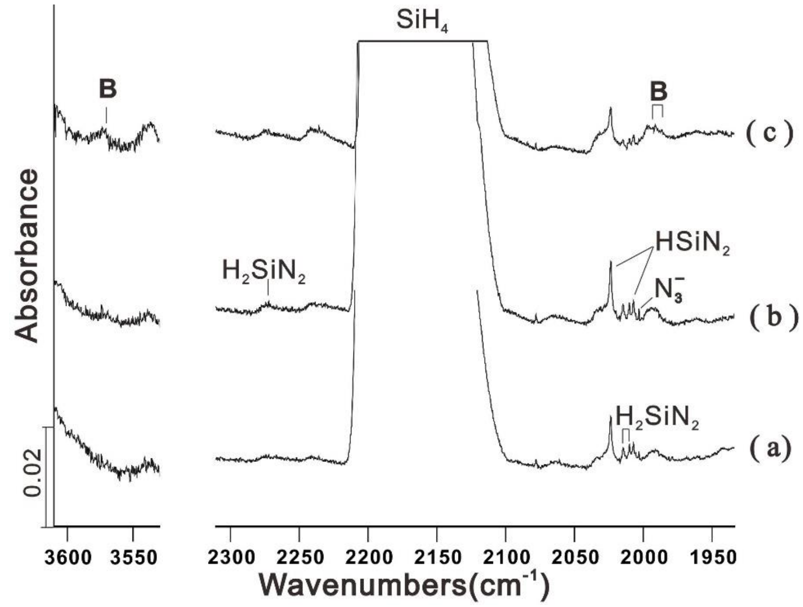

Supplementary Figure 18. Infrared spectra of the Tesla coil discharge of 10% SiH_4 reactions with different concentrations of H_2 in excess solid N_2 at 4 K after annealing to 33K. (a) 10% SiH_4/NN; (b) 10% SiH_4 + 5% H_2/NN; (c) 10% SiH_4 + 10% H_2/NN. **B**: $H_2SiNN(H_2)$

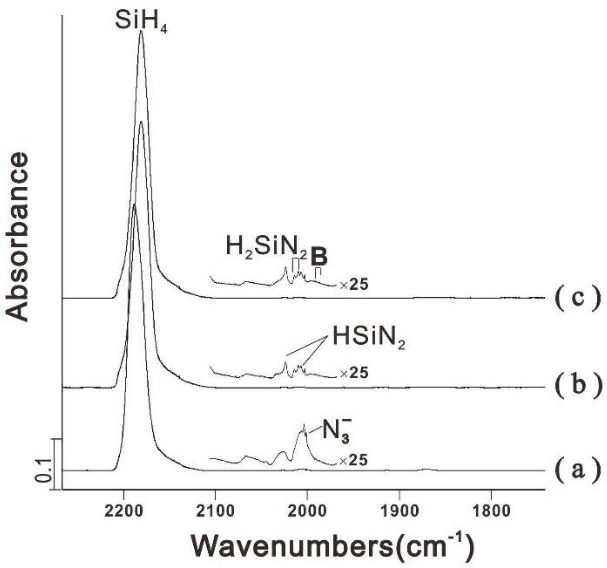

Supplementary Figure 19. Infrared spectra of the Tesla coil discharge of 10% SiH_4 reactions with 10% H_2 in excess solid $^{14}N_2$ at 4 K (a) after codeposition for 120 min; (b) annealing to 25K (c)λ > 220 nm irradiation for 10 min. **B**: $H_2SiNN(H_2)$

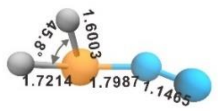

TS 1

Frequency	Infrared
-876.8	30
140.5	81
261.5	1
355.5	50
552.5	45
651.7	98
908.6	172
1696.5	143
1895.3	380

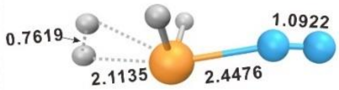

TS 2

Frequency	Infrared
-115.3	10
107.9	0
110.9	2
273.6	4
285.5	2
304.3	8
332.6	0
678.2	22
701.0	0
850.0	90
976.6	57
2062.6	155
2071.3	234
2418.1	26
4075.3	10

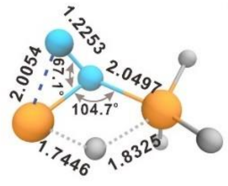

TS 3

Frequency	Infrared
-569.8	226
189.8	0
214.0	14
228.5	3
398.3	112
470.9	74
538.8	160
743.1	51
794.6	275
877.0	330
923.7	59
970.2	82
1078.4	28
1402.1	82
1571.6	111

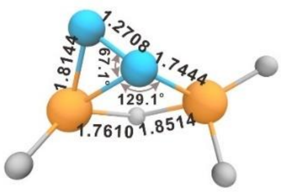

TS 4

Frequency	Infrared
-1007.9	670
165.7	3
231.3	26
431.3	2
477.2	12
528.1	11
557.0	27
618.0	56
654.8	35
723.7	54
743.6	39
897.5	43
996.0	173
1323.3	34
1469.2	96
2097.3	312
2140.6	124

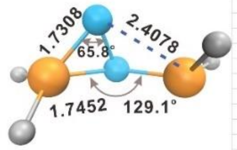

TS 5

Frequency	Infrared
-329.7	61
127.1	54
244.8	109
458.2	38
541.6	41
594.1	9
646.4	47
667.7	36
674.3	5
738.1	60
893.7	55
923.8	113
979.7	2
1041.3	131
2177.4	86
2224.8	104
2259.8	67

Supplementary Figure 20. Transition state structure (TS1-TS5) ($^1A_1$, C_1) for the reaction of **B** + SiNN → **A** + N_2 (Figure 5) and its frequencies (cm^{-1}) obtained at the B3LYP/6-311++G(3df, 3pd) level of theory.

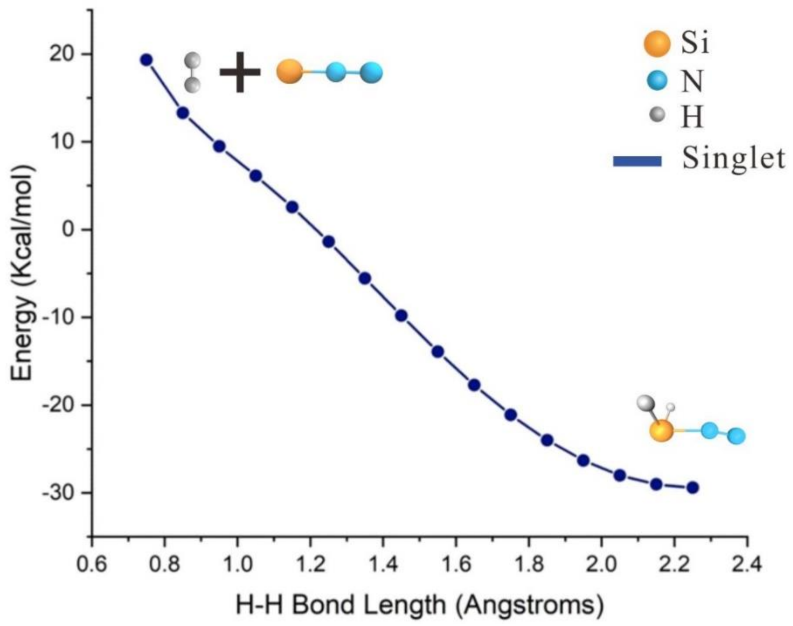

Supplementary Figure 21. Potential Energy Surface of H_2SiN_2 from H_2 and SiNN(^{1}A) on the H-H bond length from 0.6 to 2.4 Ǻ at the B3LYP/6-311++G(3df, 3pd) level of theory.

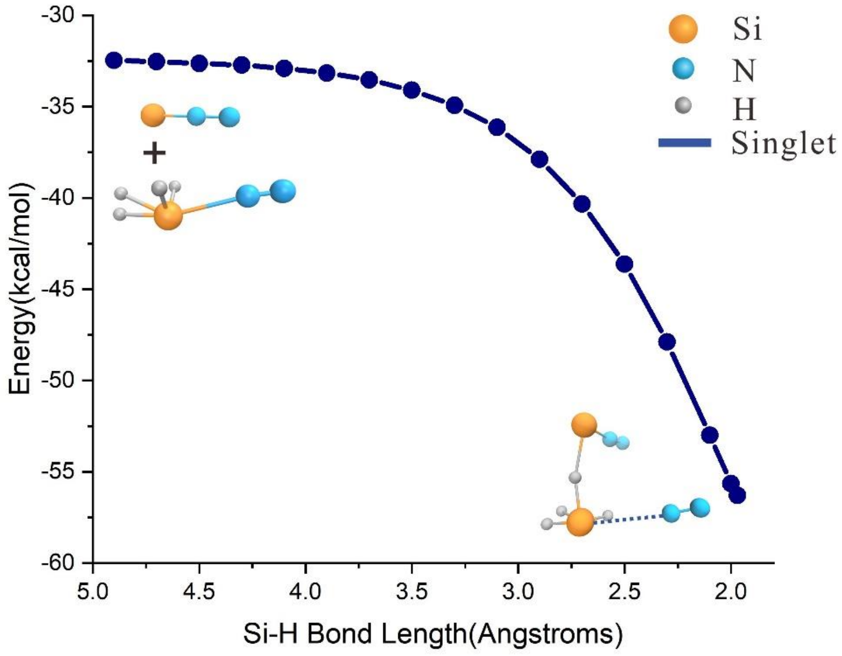

Supplementary Figure 22. Potential Energy Surface of L1 from **B** and SiNN on the H-Si bond length from 1.97 to 5.0 Ǻ at the B3LYP/6-311++G(3df, 3pd) level of theory.

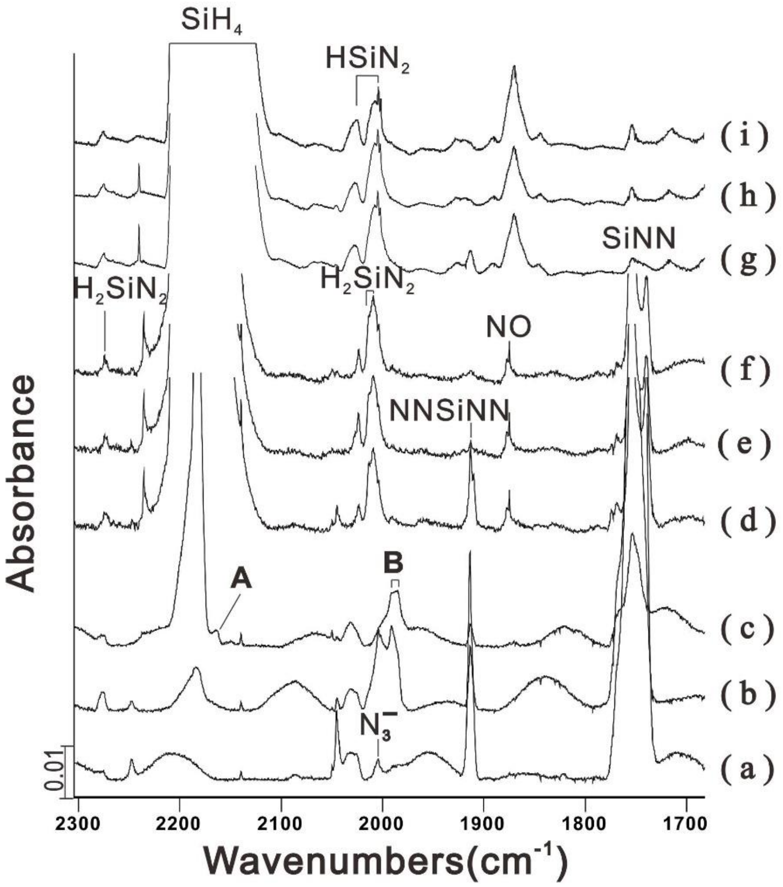

Supplementary Figure 23. Infrared spectra of different experiments to produce H_2SiN_2 and B in excess solid nitrogen at 4 K: (a) codeposition of Si + 10% H_2 for 120 min; (b) after λ > 300 nm irradiation for 10 min; (c) after λ > 220 nm irradiation for 10 min; (d) codeposition of the laser-ablated Si + 10% SiH_4 for 120 min; (e) after λ > 300 nm irradiation for 10 min; (f) after λ > 220 nm irradiation for 10 min; (g) codeposition of the Tesla coil discharge of 10% SiH_4 + 10% H_2 for 120 min; (h) after λ > 300 nm irradiation for 10 min; (i) after λ > 220 nm irradiation for 10 min. **A**: $H_2Si(\mu\text{-}N)_2SiH_2$; **B**: $H_2SiNN(H_2)$

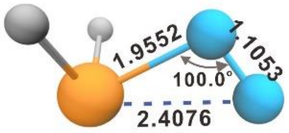

TS 6

Frequency	Infrared
-541.8	162
138.2	0
512.0	58
760.3	2
789.2	65
924.2	106
2039.0	168
2062.2	192
2228.5	140

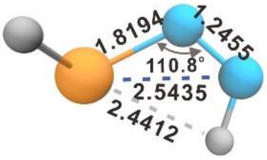

TS 7

Frequency	Infrared
-393.1	39
255.1	9
563.9	11
694.8	67
905.2	27
1475.8	79
1523.2	70
2036.4	201
2722.5	219

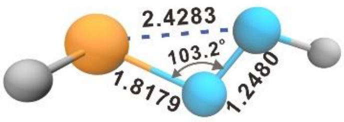

TS 8

Frequency	Infrared
-376.8	14
307.7	0
584.8	10
678.9	10
909.6	131
1450.5	38
1557.0	13
2024.8	225
3184.9	25

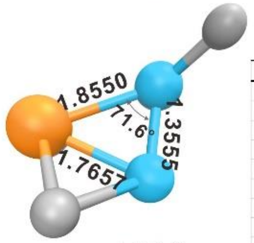

TS 9

Frequency	Infrared
-1079.3	402
563.9	57
724.4	36
888.6	102
994.8	7
1181.1	36
1300.4	27
1625.7	59
3403.4	1

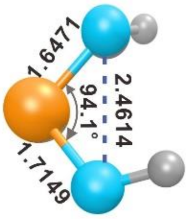

TS 10

Frequency	Infrared
-459.5	92
437.9	66
513.2	186
674.8	121
803.3	30
852.6	81
940.4	21
3475.5	20
3521.8	38

Supplementary Figure 24. Transition state structure (TS6 – TS10) ($^1A_1$, C_1) for the reaction of SiNN + H_2 → **C** (Figure 6) and its frequencies (cm^{-1}) obtained at the B3LYP/6-311++G(3df, 3pd) level of theory.

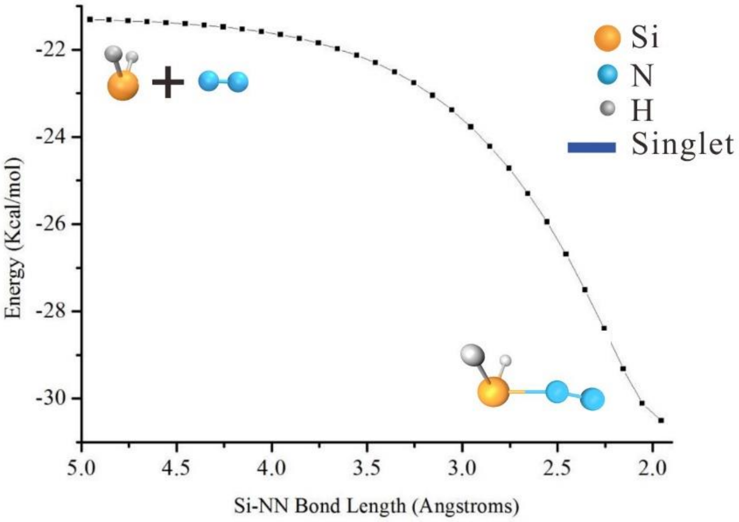

Supplementary Figure 25. Potential Energy Surface of H_2SiN_2 from H_2Si and NN on the Si-NN bond length from 1.955 to 4.955Ǻ at the B3LYP/6-311++G(3df, 3pd) level of theory.

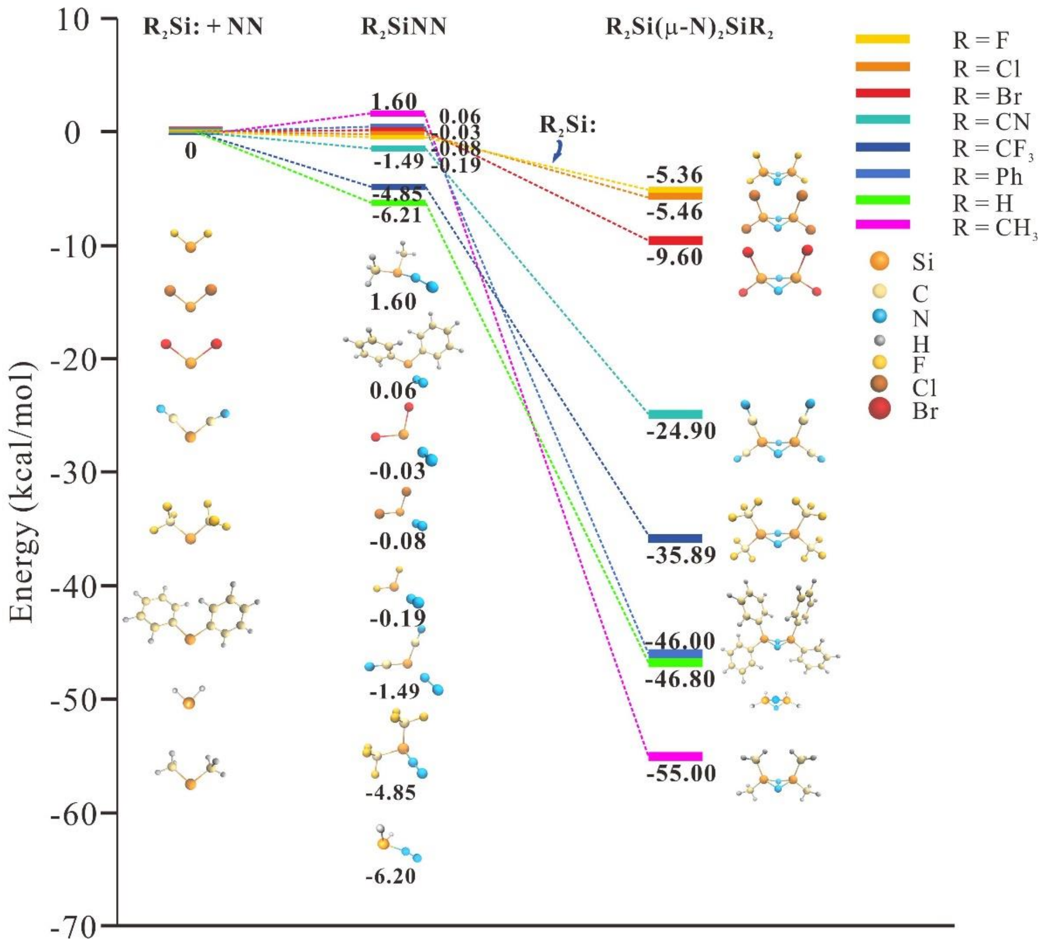

Supplementary Figure 26. Structures and zero-point energies of R_2Si:, R_2SiNN and $R_2Si(\mu$-N$)_2SiR_2$ (R=F, Cl, Br, CN, CF_3, Ph, H and CH_3). Calculated at B3LYP/6-311++g (3df, 3pd) level.

R	ΔE(R_2SiNN)[a]	ΔE($R_2Si(\mu$-N$)_2SiR_2$) [b]	ΔE_{ST}[c]
F	-0.19	-5.36	73.4
Cl	-0.08	-5.46	52.8
Br	-0.03	-9.60	47.8
CN	-1.49	-24.90	30.3
CF_3	-4.85	-35.89	27.9
Ph	0.06	-46.00	25.5
H	-6.21	-46.80	20.7
CH_3	1.60	-55.00	26.9

[a] ΔE(R_2SiNN) = E(R_2SiNN) - E(R_2Si) - E(NN); [b]ΔE($R_2Si(\mu$-N$)_2SiR_2$) = E($R_2Si(\mu$-N$)_2SiR_2$) – 2*E(R_2Si) - E(NN); [c] ΔE_{ST} is the singlet–triplet energy gap for complex R_2Si; E (kcal/mol) is the zero-point energy of the corresponding complex optimized at B3LYP/6-311++g (3df, 3pd) level.

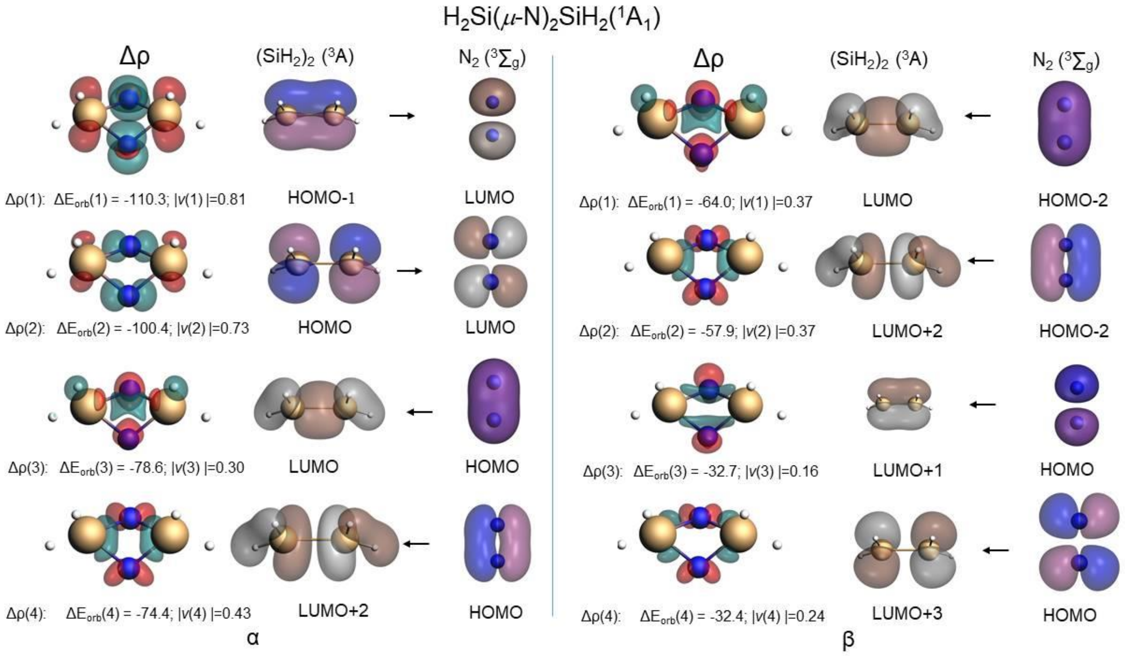

Supplementary Figure 27. Using $(SiH_2)_2{}^{2-}$ and $N_2{}^{2-}$ as interacting fragment and the shape of the deformation densities, $\Delta\rho_{(1)\text{-}(4)}$ of $H_2Si(\mu\text{-}N)_2SiH_2$ corresponding to $\Delta E_{orb(1)}$-$\Delta E_{orb(4)}$ and the associated fragment orbitals at the meta-Hybrid/M06-2X/TZP level. Isosurface values are 0.004 a. u. The eigenvalues $|v_n|$ give the size of the charge migration in e. The direction of the charge flow of the deformation densities is red→green.

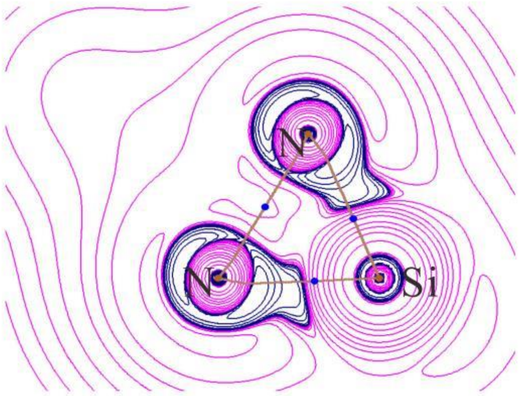

Supplementary Figure 28. Laplacian distribution of the charge density of electron localization function (ELF) for $H_2Si(\mu\text{-}N)_2SiH_2$ in the plane Si-N-N.

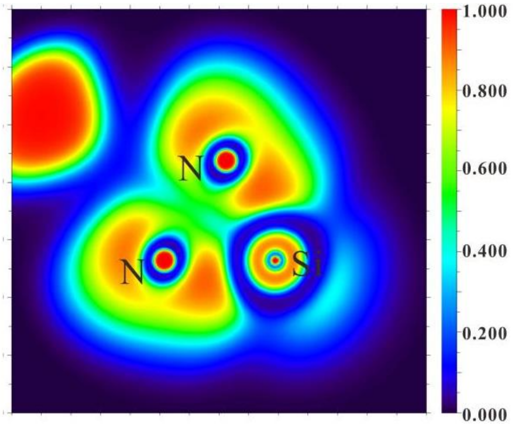

Supplementary Figure 29. Isosurface map of electron localization function (ELF) for $H_2Si(\mu$-$N)_2SiH_2$ in the plane Si-N-N.

Supplementary Tables

Supplementary Table 1. Infrared absorptions (cm^{-1}) observed for products of the reactions of Si atoms with H_2 molecules in solid N_2

Mode assignment	$H_2/^{14}N_2$	$D_2/^{14}N_2$	$HD/^{14}N_2$	$H_2+D_2/^{14}N_2$	$H_2/^{15}N_2$	$H_2/^{14}N_2+^{15}N_2$	$D_2/^{15}N_2$
**A** $H_2Si(\mu\text{-}N)_2SiH_2$ ($^1A_1$, C_{2v})							
$H_2Si\text{-}SiH_2$ stretch, b_2	cover	1586.1	1580.1, 1575.8	1586.1,1577.2	cover	cover	1586.1
$H_2Si\text{-}SiH_2$ stretch, a_1	2165.3	1551.3	2167.4, 2162.4, 1556.8, 1550.5	2165.3, 2163.0 1551.3, 1549.2	2165.3	2165.3	1551.3
$2SiH_2$ bend, b_2	937.8	679.2	847.5, 845.3	946.4, 937.8	937.2	937.8, 937.2	679.2
Si(NN)Si ring, b_2	857.7	855.3	cover	cover	cover	857.7	834.7
**B** $H_2SiNN(H_2)$ ($^1A_1$, C_s)							
H-H stretch, a'	3569.3	2530.6	3110.3, 3102.4	3569.3	3569.3	3569.3	2530.6
SiH_2 stretch, a''	1991.6	1425.9	1987.2, 1984.5	1991.2, 1425.2	1991.6	1991.6	1425.9
SiH_2 stretch, a'	1983.6	1421.8	1425.3	1986.8, 1421.2	1983.6	1983.6	1421.8
H-H twist, a'	1097.5	804.4	977.0	1195.8	1097.5	1097.5	804.4
SiH_2 scissor, a'	952.4	684.4	830.0, 828.6	952.4, 714.9, 684.4	950.9	952.4, 950.9	684.4
**C** HNSiNH ($^1A_1$, C_2)							
NH stretch, υ_{as}	3564.8	2662.5	3564.8, 2662.5	3564.8, 2662.5	3556.2	3564.8, 3556.2	2647.3
NSiN stretch, υ_{as}	1197.6	1164.8	1197.6, 1178.4, 1164.8	1197.6, 1178.4, 1164.8	1175.9	1197.6, 1175.9	1143.2

Supplementary Table 2. Observed and Calculated Fundamental Frequencies (cm^{-1}) of $H_2Si(\mu\text{-}N)_2SiH_2$ (C_{2v}, $^1A_1$) in $^{14}N_2$ matrix at B3LYP/6-311++G(3df,3pd), CCSD(T)/aug-cc-pVDZ level and CCSD(T)/aug-cc-pVTZ level.

Approximate mode description and mode symmetry	B3LYP/6-311++G(3df,3pd)	CCSD(T)/aug-cc-pVDZ	CCSD(T)/aug-cc-pVTZ	Obs.
**A1** $H_2Si(\mu\text{-}N)_2SiH_2$				
H_2Si-SiH_2 stretch, a_1	2280.5(66)	2277.7	2252.1	
H_2Si-SiH_2 stretch, b_2	2277.3(137)	2275.3	2242.5	Cover by SiH_4(2183)
H_2Si-SiH_2 stretch, a_1	2240.1(161)	2230.9	2235.5	2165.3
H_2Si-SiH_2 stretch, b_2	2229.6(46)	2221.3	2192.3	
$2SiH_2$ scissor, a_1	1025.8(25)	987.3	1003.6	
$2SiH_2$ scissor, b_2	999.9(308)	964.4	984.2	937.8
Si(NN)Si ring, b_2	953.7(170)	874.3	849.4	857.7
Si(NN)Si ring, a_1	850(14)	806.7	824.0	
Si(NN)Si deform, a_2	837.2(0)	790.5	791.7	
$2SiH_2$ wag, b_1	827.3(113)	787.9	763.2	
$2SiH_2$ twist, a_2	704.5(0)	682.8	694.0	
$2SiH_2$ rock, a_1	701.2(84)	671.3	663.5	
$2SiH_2$ rock, b_2	668.4(17)	646.1	661.8	
Si(NN)Si deform, b_1	658.4(3)	642.1	632.5	
$2SiH_2$ twist, b_1	533.1(4)	529.1	544.5	
$2SiH_2$ twist, a_2	507(0)	506.7	528.0	
**A2** $D_2Si(\mu\text{-}N)_2SiD_2$				
D_2Si-SiD_2 stretch, a_1	1647.2(65)	1645.1	1630.8	
D_2Si-SiD_2 stretch, b_2	1644.4(76)	1642.0	1619.0	1586.1
D_2Si-SiD_2 stretch, a_1	1610.5(68)	1601.7	1600.3	1551.3
D_2Si-SiD_2 stretch, b_2	1601.1(62)	1593.1	1572.8	
$2SiD_2$ scissor, a_1	708.2(0)	741.6	795.3	
$2SiD_2$ scissor, b_2	714.8(79)	689.5	703.4	679.2
Si(NN)Si ring, b_2	952.2(287)	872.6	848.0	855.3
Si(NN)Si ring, a_1	876.6(24)	818.0	689.3	
Si(NN)Si deform, a_2	799.1(0)	689.5	715.8	
$2SiD_2$ wag, b_1	718.9(81)	694.3	694.3	
$2SiD_2$ twist, a_2	553.7(0)	678.2	554.0	
$2SiD_2$ rock, a_1	556.7(64)	545.9	550.2	
$2SiD_2$ rock, b_2	504.5(13)	487.3	499.1	
Si(NN)Si deform, b_1	551.8(10)	533.3	541.9	
$2SiD_2$ twist, b_1	404(2)	399.5	375.6	
$2SiD_2$ twist, a_2	359.1(0)	359.9	416.2	
**A3** $D_2Si(\mu\text{-}N)_2SiH_2$				
SiH stretch, b_2	2279.9(105)	2275.9	2242.3	Cover by SiH_4(2183)
SiH stretch, b_2	2236.2(101)	2226.6	2192.7	2163.0
SiD stretch, b_2	1645.8(70)	1643.9	1630.5	1577.2
SiD stretch, b_2	1605.8(65)	1597.1	1599.8	1549.2
SiH_2 scissor, b_2	1013.4(141)	976.4	995.8	946.4
SiD_2 scissor, b_2	713.5(28)	686.4	697.9	
Si(NN)Si ring, b_2	953.4(246)	873.8	849.0	Cover by SiH_2D_2
Si(NN)Si ring, b_2	862.2(30)	803.7	708.7	
Si(NN)Si deform, a	834.1(31)	795.8	810.2	

SiH_2 wag, a	768.1(57)	727.7	779.3	
SiH_2 twist, a	668.3(9)	646.1	697.9	
SiH_2 rock, b_2	684.2(67)	663.8	640.6	
SiD_2 rock, b_2	529.4(30)	515.2	523.7	
Si(NN)Si deform, a	562.2(8)	548.3	564.3	
$2SiH_2$ twist, a	512.4(0)	508.0	523.5	
$2SiD_2$ twist, a	381.4(1)	380.9	393.4	
**A4** DHSi(μ-N)$_2$SiHD				
H_2Si-SiH_2 stretch, a_1	2241.6(179)	2231.9	2240.5	2167.4
H_2Si-SiH_2 stretch, b_2	2231.6(27)	2222.6	2193.5	
D_2Si-SiD_2 stretch, a_1	1643.2(22)	1639.7	1615.2	
D_2Si-SiD_2 stretch, b_2	1639.7(117)	1636.3	1611.2	1575.8
2SiHD scissor, a_1	932.5(4)	890.6	902.8	
2SiHD scissor, b_2	891.6(76)	850.6	892.0	847.5
Si(NN)Si ring, b_2	958.4(384)	891.4	836.8	Cover by SiH_2D_2
Si(NN)Si ring, a_1	841.7(24)	787.7	747.3	
Si(NN)Si deform, a_2	809.2(0)	752.9	736.4	
2SiHD wag, b_1	758.1(104)	736.0	762.3	
2SiHD twist, a_2	661.1(0)	642.4	573.5	
2SiHD rock, a_1	657.3(1)	641.9	647.1	
2SiHD rock, b_2	578.4(68)	563.3	546.9	
Si(NN)Si deform, b_1	551.6(2)	531.8	630.2	
2SiHD twist, b_1	420.5(0)	409.8	434.0	
2SiHD twist, a_2	413(0)	403.9	429.9	
**A5** HDSi(μ-N)$_2$SiDH				
H_2Si-SiH_2 stretch, a_1	2280.9(42)	2276.9	2247.3	Cover by SiH_4(2183)
H_2Si-SiH_2 stretch, b_2	2278.2(166)	2274.1	2241.2	Cover by SiH_4(2183)
D_2Si-SiD_2 stretch, a_1	1614.4(108)	1606.9	1617.5	1556.8
D_2Si-SiD_2 stretch, b_2	1605.6(23)	1598.6	1578.4	
2SiHD scissor, a_1	943.1(61)	876.8	917.8	
2SiHD scissor, b_2	895.8(72)	860.5	886.7	845.3
Si(NN)Si ring, b_2	954.7(306)	905.7	847.8	Cover by SiH_2D_2
Si(NN)Si ring, a_1	826.3(1)	770.7	779.3	
Si(NN)Si deform, a_2	818.7(0)	766.4	758.6	
2SiHD wag, b_1	783.7(98)	766.0	748.0	
2SiHD twist, a_2	660.9(0)	631.1	597.2	
2SiHD rock, a_1	602.3(53)	589.6	639.5	
2SiHD rock, b_2	544.5(33)	527.1	537.5	
Si(NN)Si deform, b_1	610.7(0)	590.4	585.3	
2SiHD twist, b_1	463.7(9)	398.2	474.3	
2SiHD twist, a_2	397.5(0)	400.6	412.5	
**A6** HDSi(μ-N)$_2$SiHD				
SiH stretch, b_2	2279.6(100)	2276.9	1578.7	1550.5
SiH stretch, b_2	2236.6(108)	2274.1	907.6	
SiD stretch, b_2	1641.5(67)	1606.9	888.0	845.3
SiD stretch, b_2	1609.9(67)	1598.6	842.3	Cover by SiH_2D_2
2SiHD scissor, b_2	936.1(42)	883.6	769.4	
2SiHD scissor, b_2	896.3(81)	857.1	737.4	
Si(NN)Si ring, b_2	957.2(335)	898.3	753.8	
Si(NN)Si ring, b_2	832.4(3)	777.3	588.2	
Si(NN)Si deform, a	815.5(3)	766.4	646.5	
2SiHD wag, a	768.5(98)	742.9	542.6	

2SiHD twist, a	669.8(1)	650.5	602.3	
2SiHD rock, b_2	590.8(67)	577.9	458.3	
2SiHD rock, b_2	548.2(13)	528.8	419.8	
Si(NN)Si deform, a	627.3(1)	605.0	1578.7	1550.5
2SiHD twist, b_2	451.4(5)	440.8	907.6	
2SiHD twist, a	402.5(1)	401.8	888.0	845.3

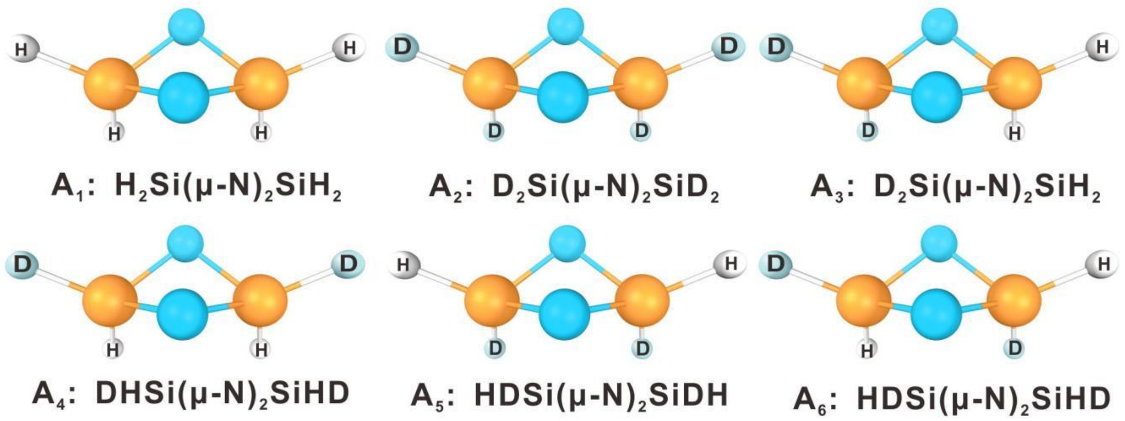

Supplementary Table 3. Observed and Calculated Fundamental Frequencies (cm^{-1}) of $H_2Si(\mu\text{-}^{15}N)_2SiH_2$ (C_{2v}, $^1A_1$) in ^{15}N matrix at B3LYP/6-311++G(3df,3pd), CCSD(T)/aug-cc-pVDZ level and CCSD(T)/aug-cc-pVTZ level.

Approximate mode description and mode symmetry	B3LYP/6-311++G(3df,3pd)	CCSD(T)/aug-cc-pVDZ	CCSD(T)/aug-cc-pVTZ	Obs.
$H_2Si(\mu\text{-}^{15}N)_2SiH_2$				
$H_2Si\text{-}SiH_2$ stretch, a_1	2280.5(66)	2277.7	2252.1	
$H_2Si\text{-}SiH_2$ stretch, b_2	2277.3(137)	2275.2	2242.5	cover
$H_2Si\text{-}SiH_2$ stretch, a_1	2240.1(161)	22308	2235.5	2165.3
$H_2Si\text{-}SiH_2$ stretch, b_2	2229.6(46)	2221.2	2192.3	
$2SiH_2$ scissor, a_1	1024.8(24)	986.4	1003.3	
$2SiH_2$ bend, b_2	999.7(287)	964.4	984.2	937.2
Si(NN)Si ring, b_2	933.5(180)	855.7	831.3	cover
Si(NN)Si ring, a_1	832.6(14)	804.3	822.2	
Si(NN)Si deform, a_2	819.8(0)	774.0	782.7	
$2SiH_2$ wag, b_1	824.7(110)	774.2	747.2	
$2SiH_2$ twist, a_2	699.2(0)	664.2	692.5	
$2SiH_2$ rock, a_1	699.5(83)	681.3	661.8	
$2SiH_2$ rock, b_2	668.4(17)	645.8	652.6	
Si(NN)Si deform, b_1	644.4(3)	629.0	620.5	
$2SiH_2$ twist, b_1	530.1(5)	525.2	540.1	
$2SiH_2$ twist, a_2	507(0)	506.4	527.5	
$D_2Si(\mu\text{-}^{15}N)_2SiD_2$				
$D_2Si\text{-}SiD_2$ stretch, a_1	1646.6(66)	1645.1	1630.8	
$D_2Si\text{-}SiD_2$ stretch, b_2	1643.4(75)	1642.0	1619.0	1586.1
$D_2Si\text{-}SiD_2$ stretch, a_1	1609.6(68)	1601.7	1600.2	1551.3
$D_2Si\text{-}SiD_2$ stretch, b_2	1600.1(62)	1593.0	1572.8	
$2SiD_2$ scissor, a_1	704.7(0)	673.8	677.2	
$2SiD_2$ scissor, b_2	714.1(75)	688.5	702.1	679.2
Si(NN)Si ring, b_2	932.3(282)	854.9	831.1	834.7
Si(NN)Si ring, a_1	861.3(23)	804.9	679.8	
Si(NN)Si deform, a_2	776.1(0)	720.6	695.6	
$2SiD_2$ wag, b_1	706.7(81)	682.8	677.2	
$2SiD_2$ twist, a_2	553.9(0)	533.3	552.0	
$2SiD_2$ rock, a_1	554.5(62)	533.3	549.6	
$2SiD_2$ rock, b_2	504.7(13)	487.3	499.1	
Si(NN)Si deform, b_1	546(8)	533.2	535.0	
$2SiD_2$ twist, b_1	401.7(1)	391.5	413.7	
$2SiD_2$ twist, a_2	359.6(0)	359.9	375.6	

Supplementary Table 4. Observed and Calculated Fundamental Frequencies (cm^{-1}) of $H_2SiNN(H_2)$ (Cs, $^1A_1$) at B3LYP/6-311++G(3df,3pd), CCSD(T)/aug-cc-pVDZ level and CCSD(T)/aug-cc-pVTZ level.

Approximate mode description and mode symmetry	B3LYP/6-311++G(3df,3pd)	CCSD(T)/aug-cc-pVDZ	CCSD(T)/aug-cc-pVTZ	Obs.
$H_2SiNN(H_2)$				
H-H stretch, a'	3662.8(86)	3733.3	3566.6	3569.3
N-N stretch, a'	2445.8(0)	2320.9	2344.0	
SiH_2 stretch, a''	2069.5(215)	2034.4	2002.4	1991.6
SiH_2 stretch, a'	2061.5(143)	2024.8	1992.0	1983.6
H-H twist, a'	1154(50)	1129.3	1217.8	1097.5
SiH_2 scissor, a'	986.1(78)	982.2	997.5	952.4
deform, a''	788(4)	749.8	798.5	
deform, a'	742.7(27)	728.8	758.0	
H-H rock, a'	466.1(10)	470.2	627.7	
deform, a''	446.9(1)	446.0	387.4	
deform, a''	227.8(3)	226.1	293.0	
deform, a'	218.8(4)	197.9	283.1	
deform, a''	71.3(0)	75.9	249.4	
deform, a'	65.2(1)	74.2	218.4	
deform, a'	48.9(1)	50.7	60.7	
$D_2SiNN(D_2)$				
D-D stretch, a'	2591.4(43)	2641.2	2523.3	2530.6
N-N stretch, a'	2445.8(0)	2320.6	2344.0	
SiD_2 stretch, a''	1491.5(110)	1464.3	1441.8	1425.9
SiD_2 stretch, a'	1479.3(76)	1454.5	1430.7	1421.8
D-D twist, a'	821.1(26)	803.8	864.7	804.4
SiD_2 scissor, a'	707.7(39)	704.5	716.0	684.4
deform, a''	560.3(2)	533.1	566.9	
deform, a'	535(16)	526.6	549.4	
D-D rock, a'	338.2(5)	336.1	444.3	
deform, a''	316.2(1)	320.1	293.2	
deform, a''	181.1(2)	166.8	283.1	
deform, a'	179.9(2)	158.2	268.4	
deform, a''	66(0)	74.2	181.9	
deform, a'	60.5(1)	66.8	165.8	
deform, a'	47.4(0)	51.1	59.8	
$D_2SiNN(H_2)$				
H-H stretch, a'	3662.6(85)	3733.2	3566.4	3570.1
N-N stretch, a'	2445.8(0)	2320.6	2344.0	
SiD_2 stretch, a''	1491.6(111)	1464.4	1441.9	1425.2
SiD_2 stretch, a'	1479.9(77)	1454.9	1431.4	1421.2
H-H twist, a'	1134.2(39)	1113.7	1207.0	1095.4
SiD_2 scissor, a'	714.2(45)	711.5	736.9	714.9
deform, a''	653.8(2)	617.9	721.1	
deform, a'	590.1(8)	575.1	596.9	
H-H rock, a'	461.6(16)	454.4	528.1	
deform, a''	415.6(1)	428.3	385.9	
deform, a''	207.9(4)	190.3	285.2	

deform, a'	200.6(3)	184.3	282.2	
deform, a''	69.2(0)	74.7	214.1	
deform, a'	63.4(1)	69.9	200.6	
deform, a'	48.9(1)	51.9	60.0	
$H_2SiNN(D_2)$				
D-D stretch, a'	2591.7(45)	2641.4	2523.5	2530.6
N-N stretch, a'	2445.8(0)	2320.6	2344.0	
SiH_2 stretch, a''	2069.5(215)	2034.2	2002.3	1991.2
SiH_2 stretch, a'	2061.2(141)	2024.6	1991.8	1986.8
D-D twist, a'	983.2(50)	981.2	996.4	950.9
SiH_2 scissor, a'	878.3(72)	848.4	894.6	Cover by SiH_2D_2
deform, a''	725.1(4)	696.3	712.8	
deform, a'	663.5(17)	659.0	700.7	
D-D rock, a'	338.7(5)	348.7	478.5	
deform, a''	335.4(1)	325.8	293.6	
deform, a''	200.1(2)	199.2	287.4	
deform, a'	185.6(2)	163.4	268.6	
deform, a''	67.7(0)	75.4	222.9	
deform, a'	62.4(1)	70.9	173.6	
deform, a'	47.5(0)	50.5	60.6	
HDSiNN(HD)				
H-D stretch, a'	3180.7(86)	3239.7	3088.9	3110.3
N-N stretch, a'	2445.8(0)	2320.6	2344.0	
SiHD stretch, a''	2065.2(180)	2024.3	2001.9	1987.2
SiHD stretch, a'	1485.7(92)	1463.1	1433.3	1425.3
H-D twist, a'	977.8(43)	963.2	1073.3	Cover by SiH_4(936)
SiHD scissor, a'	860.1(55)	860.1	869.5	830.0
deform, a''	726.5(13)	711.9	769.1	
deform, a'	567.2(8)	542.1	634.3	
H- D rock, a'	402.8(8)	416.2	484.1	
deform, a''	380.8(2)	367.4	323.8	
deform, a''	197.8(3)	184.4	284.2	
deform, a'	191.7(2)	173.1	278.2	
deform, a''	68.4(0)	74.5	211.3	
deform, a'	62.3(1)	69.8	187.4	
deform, a'	48.1(1)	50.6	60.4	
HDSiNN(DH)				
D-H stretch, a'	3172(48)	3234.1	3088.9	3102.4
N-N stretch, a'	2445.8(0)	2320.6	2344.0	
SiHD stretch, a''	2065.3(180)	2024.4	1991.9	1984.5
SiHD stretch, a'	1486(92)	1463.4	1440.5	1425.3
D-H twist, a'	1028.7(31)	1001.3	1073.1	977.0
SiHD scissor, a'	860.8(59)	858.0	874.2	828.6
deform, a''	751.8(9)	725.5	766.6	
deform, a'	595.5(22)	582.4	645.0	
D-H rock, a'	384.3(4)	375.9	478.5	
deform, a''	358.9(0)	368.9	323.9	
deform, a''	199.8(3)	193.2	284.3	
deform, a'	192.2(3)	170.5	278.0	
deform, a''	68.4(0)	74.7	213.3	
deform, a'	63(1)	69.8	182.9	
deform, a'	48.1(1)	51.9	60.2	
$H_2Si^{15}N^{15}N(H_2)$				

H-H stretch, a'	3662.8(86)	3733.3	3566.5	3569.3
^{15}N-^{15}N stretch, a'	2363.1(0)	2242.5	2265.0	
SiH_2 stretch, a''	2069.5(215)	2034.3	2002.4	1991.6
SiH_2 stretch, a'	2061.5(143)	2024.7	1992.0	1983.6
H-H twist, a'	1154(50)	1129.2	1217.8	1097.5
SiH_2 scissor, a'	986.1(78)	982.2	997.5	952.4
deform, a''	788(4)	749.8	798.5	
deform, a'	742.7(27)	728.7	758.0	
H-H rock, a'	466(10)	470.2	627.8	
deform, a''	446.9(1)	445.9	387.1	
deform, a''	226.5(3)	225.7	286.5	
deform, a'	217.1(4)	196.8	274.4	
deform, a''	69.6(0)	74.4	247.4	
deform, a'	63.4(1)	72.7	216.7	
deform, a'	47.9(0)	48.9	59.6	
$D_2Si^{15}N^{15}N(D_2)$				
D-D stretch, a'	2591.4(43)	2641.2	2523.3	2530.6
^{15}N-^{15}N stretch, a'	2363.1(0)	2242.5	2265.0	
SiD_2 stretch, a''	1491.5(111)	1464.3	1441.8	1425.9
SiD_2 stretch, a'	1479.3(76)	1454.4	1430.7	1421.8
D-D twist, a'	821.1(26)	803.8	864.7	804.4
SiD_2 scissor, a'	707.7(39)	704.5	716.0	684.4
deform, a''	560.3(2)	533.1	566.9	
deform, a'	534.9(16)	526.6	549.3	
D-D rock, a'	338(5)	336.0	444.3	
deform, a''	316.1(1)	320.0	288.2	
deform, a''	178.8(2)	166.1	273.6	
deform, a'	178.1(2)	156.7	263.6	
deform, a''	64.6(0)	72.8	181.7	
deform, a'	59(1)	65.4	165.5	
deform, a'	46.4(0)	49.5	58.7	

Supplementary Table 5. Observed and Calculated Fundamental Frequencies (cm^{-1}) of HNSiNH (C_2, $^1A_1$) at B3LYP/6-311++G(3df,3pd), CCSD(T)/aug-cc-pVDZ level and CCSD(T)/aug-cc-pVTZ level.

Approximate mode description and mode symmetry	B3LYP/6-311++G(3df,3pd)	CCSD(T)/aug-cc-pVDZ	CCSD(T)/aug-cc-pVTZ	Obs.
HNSiNH				
NH str, υ_s	3671.2(13)	3561.1	3591.8	
NH str, υ_{as}	3670.5(176)	3559.9	3591.4	3564.8
NSiN str, υ_{as}	1353.1(106)	1228.1	1278.8	1197.6
NSiN str, υ_s	972.7(6)	882.7	919.2	
HN-NH sci	537.6(57)	671.8	648.1	
HN-NH rock	461(189)	608.0	584.7	
HN-NH twist	384.7(22)	440.4	440.2	
deform	325.1(20)	298.4	307.9	
DNSiND				
NH str, υ_s	2693.5(14)	2608.4	2630.7	
NH str, υ_{as}	2693.5(124)	2607.4	2630.1	2662.5
NSiN str, υ_{as}	1315.6(128)	1194.2	1245.7	1164.8
NSiN str, υ_s	921.8(1)	829.4	870.8	
HN-NH sci	421.9(42)	531.4	509.9	
HN-NH rock	355.9(91)	470.4	452.3	
HN-NH twist	294.2(7)	324.6	324.5	
deform	267.4(18)	245.4	248.9	
DNSiNH				
NH str, υ_s	3670.9(94)	3558.5	3591.5	3564.8
NH str, υ_{as}	2693.5(69)	2606.0	2630.4	2662.5
NSiN str, υ_{as}	1335.6(115)	1211.2	1263.3	1178.4
NSiN str, υ_s	945.8(6)	852.7	893.3	
HN-NH sci	510.4(95)	650.5	623.8	
HN-NH rock	383.4(57)	497.6	478.2	
HN-NH twist	349.6(66)	391.0	391.7	
deform	283.1(8)	263.3	268.1	
$H^{15}NSi^{15}NH$				
NH str, υ_s	3662.3(92)	3552.8	3583.5	
NH str, υ_{as}	3661.7(132)	3551.9	3583.0	3556.2
NSiN str, υ_{as}	1334.6(37)	1211.9	1261.5	1175.9
NSiN str, υ_s	946.3(12)	862.7	895.9	
HN-NH sci	532.9(116)	663.1	641.3	
HN-NH rock	383.5(42)	603.5	580.8	
HN-NH twist	323.7(47)	439.1	439.2	
deform	203.2(0)	297.4	307.0	
$D^{15}NSi^{15}ND$				
NH str, υ_s	2680.5(13)	2596.3	2618.8	
NH str, υ_{as}	2680.4(120)	2595.2	2618.2	2647.3
NSiN str, υ_{as}	1298.5(123)	1177.8	1228.9	1143.2
NSiN str, υ_s	895.4(1)	804.8	845.3	
HN-NH sci	417.9(41)	527.2	505.7	
HN-NH rock	293.4(7)	466.6	448.3	
HN-NH twist	266.4(17)	323.7	323.3	
deform	187.4(39)	244.4	248.0	

Supplementary Table 6. Comparison between the observed and calculated vibrational frequencies (cm^{-1}) and isotopic frequency ratios of the new products

Approximate description	frequency(cm-1)			$R_{H/D}$			$R_{14N/15N}$			$R_{H-14N/D-15N}$		
	Calc(CCSD(T))		obs	Calc(CCSD(T))		obs	Calc(CCSD(T))		obs	Calc(CCSD(T))		obs
	DZ	TZ		DZ	TZ		DZ	TZ		DZ	TZ	
A $H_2Si(\mu\text{-}N)_2SiH_2$												
HSi-SiH stretch, b_2	2275.3	2242.5	cover	1.3857	1.3851	/	1.0000	1.0000	/	1.3857	1.3851	/
HSi-SiH stretch, a_1	2230.9	2235.5	2165.3	1.3928	1.3970	1.3958	1.0000	1.0000	1.0000	1.3929	1.3970	1.3958
$2SiH_2$ bend, b_2	964.4	984.2	937.8	1.3988	1.4019	1.3807	1.0000	1.0000	1.0006	1.4008	1.4019	1.3807
Si(NN)Si ring, b_2	874.3	849.4	857.7	1.0019	1.0002	1.0028	1.0217	1.0218	/	1.0227	1.0220	1.0276
B H_2SiNN												
H-H stretch, a'	3733.3	3566.5	3569.3	1.4135	1.4134	1.4105	1.0000	1.0000	1.0000	1.4135	1.4134	1.4105
N-N stretch, a'	2320.9	2344.3	2276.7	1.0001	1.0001	1.0007	1.0350	1.0350	1.0340	1.0350	1.0350	1.0340
SiH_2 stretch, a''	2034.4	2002.4	1991.6	1.3894	1.3889	1.3967	1.0001	1.0000	1.0000	1.3894	1.3889	1.3967
SiH_2 stretch, a'	2024.8	1992.0	1983.6	1.3922	1.3923	1.3951	1.0001	1.0000	1.0000	1.3922	1.3923	1.3951
H-H twist, a'	1129.3	1217.8	1097.5	1.4050	1.4083	1.3644	1.0000	1.0000	1.0000	1.4050	1.4083	1.3644
SiH_2 scissor, a'	982.2	997.5	952.4	1.3942	1.3930	1.3916	1.0001	1.0000	1.0000	1.3942	1.3930	1.3916
C HNSiNH												
NH str, υ_{as}	3559.9	3591.4	3564.8	1.3653	1.3652	1.3389	1.0023	1.0023	1.0024	1.3717	1.3717	1.3466
NSiN str, υ_{as}	1228.1	1278.8	1197.6	1.0284	1.0266	1.0282	1.0133	1.0137	1.0186	1.0427	1.0406	1.0476

Supplementary Table 7. Infrared absorptions (cm^{-1}) observed for SiNN, NNSiNN and H_2SiN_2 in solid $^{14}N_2/^{15}N_2$

Mode assignment	$H_2/^{14}N_2$	$D_2/^{14}N_2$	$HD/^{14}N_2$	$H_2/^{15}N_2$
SiNN ($C_{\infty v}$, ^3A)				
NN str	2247.7	2247.7	2247.7	cover
NN + SiN str	1754.7	1754.7	1754.7	1697.1
NNSiNN (C_{2v}, ^1A)				
NN str	2245.1	2245.1	2245.1	1977.0
NN + SiN str	1913.3	1913.3	1913.3	1849.4
H_2SiN_2 (C_{2v}, ^1A)				
NN str, a'	2274.2	2274.2	2274.2	2198.6
SiH_2 str, a''	2013.6	1466.2	2013.3	2013.6
SiH_2 str, a'	2009.4	1460.4	1461.8	2009.4
SiH_2 sci, a'	928.8	669.9	815.1	928.2
$HSiN_2$ (C_s, $^2A''$)				
NN str, a'	2023.9	2023.9	2023.9	1957.1
SiH str, a'	2006.6	1458.9	2006.6,1458.9	2006.6

Supplementary Table 8. Electron Density of Delocalized Bonds (EDDB) of $H_2Si(\mu\text{-}N)_2SiH_2$(**A**)

complex	π/e	σ/e	Total/e
**A**	0.62	0.93	1.55

Supplementary Table 9. Observed and calculated (B3LYP/6-311++G(3df, 3pd), CCSD(T)/aug-cc-pVDZ and CCSD(T)/aug-cc-pVTZ) infrared absorptions (cm^{-1}) for products of *B* and H_2SiN_2

Approximate description	B3LYP/6-311++g(3df,3pd)			CCSD(T)/aug-cc-pVDZ			CCSD(T)/aug-cc-pVTZ			Obs.		
	$H_2SiNN(H_2)$	$H_2SiNN(H_2)$	$H_2SiNN(H_2)$	$H_2SiNN(H_2)$	H_2SiN_2	$\Delta\nu$	$H_2SiNN(H_2)$	H_2SiN_2	$\Delta\nu$	$H_2SiNN(H_2)$	H_2SiN_2	$\Delta\nu$
	3662.8(86)			3733.3			3566.6			3569.3		
N-N stretch, a'	2445.8(0)	2300.4(250)		2320.9	2310.3		2344.0	2301.6			2274.2	
SiH_2 stretch, a''	2069.5(215)	2073.8(193)	-4.34	2034.4	2035.1	-0.71	2002.4	2066.1	-63.66	1991.6	2013.6	-22.0
SiH_2 stretch, a'	2061.5(143)	2068.8(122)	-7.22	2024.8	2030.4	-5.58	1992.0	2065.6	-73.66	1983.6	2009.4	-25.8
H-H twist, a'	1154(50)			1129.2			1217.8			1097.5		
SiH_2 sci, a'	986.1(78)	956.5(70)	+29.53	982.2	980.4	+1.8	997.5	973.7	+23.78	952.4	928.8	+23.6
	788(4)	750.7(29)		749.8	681.1		798.5	755.8				
	742.7(27)	743.3(10)		728.8	620.6		758.0	710.7				
	466.1(10)	315.6(0)		470.2	182.3		627.7	232.2				
	446.9(1)	279.8(3)		446.0	158.6		387.4	197.6				
	227.8(3)	247.2(1)		226.1	134.7		292.0	177.4				
	218.8(4)			197.9			283.1					
	71.3(0)			75.9			249.4					
	65.2(1)			74.22			218.4					
	48.9(1)			50.7			60.7					

Supplementary Table 10. Observed and Calculated Fundamental Frequencies (cm^{-1}) of H_2SiN_2 in low H_2 concentration at B3LYP/6-311++G(3df,3pd), CCSD(T)/aug-cc-pVdZ and CCSD(T)/aug-cc-pVTZ level.

Approximate mode description and mode symmetry	B3LYP/6-311++G(3df,3pd)	CCSD(T)/aug-cc-pVDZ	CCSD(T)/aug-cc-pVTZ	Obs.
H_2SiN_2				
NN str, a'	2300.43(250)	2310.3	2301.59	2274.2
SiH_2 str, a''	2073.83(193)	2034.1	2066.08	2013.6
SiH_2 str, a'	2068.76(122)	2030.4	2065.63	2009.4
SiH_2 sci, a'	956.53(70)	980.4	973.69	928.8
SiH_2 wag, a'	750.7(29)	681.1	755.84	
SiH_2 twi, a''	743.33(10)	620.6	710.71	
deform, a'	315.62(0)	182.3	232.19	
deform, a''	279.81(3)	158.8	197.63	
deform	247.21(1)	134.7	177.4	
D_2SiN_2				
NN str, a'	2299.72(252)	2310.3	2301.49	2274.2
SiD_2 str, a''	1494.89(100)	1462.7	1488.24	1466.2
SiD_2 str, a'	1483.24(65)	1460.5	1482.73	1460.4
SiD_2 sci, a'	690.1(34)	705.3	700.01	669.9
SiD_2 wag, a'	584.16(18)	513.3	575.47	
SiD_2 twi, a''	564.01(4)	458.6	530.28	
deform, a'	303.27(1)	174.7	220.91	
deform, a''	270.85(2)	152.5	188	
deform	230.63(1)	134.7	177.31	
$HDSiN_2$				
NN str, a'	2300.08(251)	2310.3	2301.54	2274.2
SiHD str, a''	2071.03(158)	2031.9	2065.55	2013.3
SiHD str, a'	1489.42(81)	1461.9	1485.81	1461.8
SiHD sci, a'	836.73(51)	854.3	848.33	815.1
SiHD wag, a'	743.25(22)	654.5	735.68	
SiHD twi, a''	575.49(11)	481.9	550.47	
deform, a'	309.26(0)	178.8	227.18	
deform, a''	276.62(3)	155.2	191.95	
deform	237.29(1)	134.7	177.36	
$H_2Si^{15}N_2$				
$^{15}N^{15}N$ str, a'	2222.87(232)	2232.2	2223.81	2198.6
SiH_2 str, a''	2073.82(193)	2034.1	2066.06	2013.6
SiD_2 str, a''	2068.66(123)	2030.4	2065.63	2009.4
SiD_2 str, a'	956.17(70)	980.4	973.66	928.2
SiD_2 sci, a'	747.38(28)	679.3	753.42	
SiD_2 wag, a'	740.45(10)	619	708.57	
deform, a'	308.01(0)	177.2	225.61	
deform, a''	273.85(3)	153.9	191.78	
deform	240.27(1)	132.1	174.09	

Supplementary Table 11. Observed and Calculated Fundamental Frequencies (cm^{-1}) of HNSiNH(NN)$_n$,n=0-2 at B3LYP/6-311++G(3df,3pd) level.

	HNSiNH	HNSiNH(NN)	HNSiNH(NN)$_2$	
Optimized structures (bond lengths in Å and bond angles in degree) at B3LYP/6-311++G(3df,3pd)				
	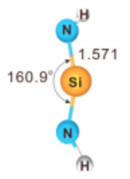	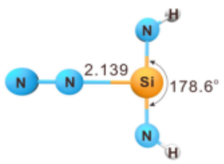	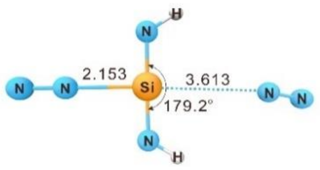	
Frequencies (cm^{-1})				
Mode description	Calculated Fundamental Frequencies (cm^{-1}) at B3LYP/6-311++G(3df,3pd)			Obs.
NH sym-str	3671.2(13)	3641.2(34)	3636.2(42)	
NH as-str	3670.5(176)	3640.5(94)	3635(70)	3564.8
NN str			2447.1(0)	
NN str		2440.4(4)	2440.4(4)	
NSiN as-str	1353.1(106)	1307.3(114)	1304(110)	1197.6
NSiN sym-str	972.7(6)	942.3(27)	938.6(32)	
	537.6(57)	613(32)	623.6(47)	
	461(189)	549.4(229)	559.9(235)	
	384.7(22)	473.3(116)	476.6(109)	
	325.1(20)	355.2(4)	355.1(2)	
		346.4(0)	353.5(0)	
		302.3(52)	300.8(57)	
		240.5(64)	242.8(59)	
		189.5(2)	189.4(1)	
		120.7(2)	122.7(3)	
		102.6(27)	91(33)	
			50.3(0)	
			42.7(0)	
			37.4(0)	
			14(0)	
			7.3(0)	

Supplementary Table 12 NBO reaults for $H_2Si(\mu\text{-}N)_2SiH_2$, $H_2SiNN(H_2)$, H_2SiNN and HNSiNH.

molecule	bond	NLMO	occ	natural charges
	Si1-H1	40% Si($s^{0.27}p^{0.72}d^{0.01}$) + 60% H(s)	1.97	Si: 1.45
	Si1-H2	41% Si($s^{0.31}p^{0.68}d^{0.01}$) + 59% H(s)	1.98	N1: -1.07
	Si1-N1	21% Si($s^{0.21}p^{0.77}d^{0.02}$) + 79% N($s^{0.29}p^{0.70}d^{0.01}$)	1.97	N2: -1.07
	Si1-N2	21% Si($s^{0.21}p^{0.77}d^{0.02}$) + 79% N($s^{0.29}p^{0.70}d^{0.01}$)	1.97	
	N1-N2	50% N($s^{0.01}p^{0.98}d^{0.01}$) + 50% N($s^{0.01}p^{0.98}d^{0.01}$)	1.94	
$H_2Si(\mu\text{-}N)_2SiH_2$	Si2-N1	21% Si($s^{0.21}p^{0.77}d^{0.02}$) + 79% N($s^{0.29}p^{0.70}d^{0.01}$)	1.97	
	Si2-N2	21% Si($s^{0.21}p^{0.77}d^{0.02}$) + 79% N($s^{0.29}p^{0.70}d^{0.01}$)	1.97	
	Si2-H3	41% Si($s^{0.31}p^{0.68}d^{0.01}$) + 59% H(s)	1.98	
	Si2-H4	40% Si($s^{0.27}p^{0.72}d^{0.01}$) + 60% H(s)	1.97	
	LP(N1)	100% N($s^{0.41}p^{0.59}$)	1.93	
	LP(N2)	100% N($s^{0.41}p^{0.59}$)	1.93	
	Si-H1	38% Si($s^{0.15}p^{0.85}$) + 62% H(s)	1.98	Si: 0.42
	Si-H2	38% Si($s^{0.15}p^{0.85}$) + 62% H(s)	1.98	N1: -0.10
	N1-N2	51% N(p) + 49% N(p)	2.00	N2: 0.09
	N1-N2	51% N($s^{0.06}p^{0.94}$) + 49% N($s^{0.06}p^{0.94}$)	2.00	
$H_2SiNN(H_2)$	N1-N2	50% N($s^{0.34}p^{0.66}$) + 50% N($s^{0.34}p^{0.66}$)	2.00	
	H3-H4	52% N($s^{0.99}p^{0.01}$) + 48% N(s)	1.82	
	LP(Si)	100% Si($s^{0.69}p^{0.31}$)	1.93	
	LP(N1)	100% N($s^{0.62}p^{0.38}$)	1.95	
	LP(N2)	100% N($s^{0.62}p^{0.38}$)	1.99	
	Si-H1	38% Si($s^{0.14}p^{0.85}d^{0.01}$) + 62% H(s)	1.94	Si: 0.42
	Si-H2	38% Si($s^{0.14}p^{0.85}d^{0.01}$) + 62% H(s)	1.94	N1: -0.11
	Si-N1	12% Si($s^{0.06}p^{0.93}d^{0.01}$) + 88% N($s^{0.59}p^{0.41}$)	1.99	N2: 0.09
	N1-N2	53% N($s^{0.19}p^{0.81}$) + 47% N($s^{0.15}p^{0.85}$)	2.00	
H_2SiNN	N1-N2	52% N($s^{0.24}p^{0.76}$) + 48% N($s^{0.25}p^{0.75}$)	2.00	
	N1-N2	55% N(p) + 45% N($p^{0.99}p^{0.01}$)	2.00	
	LP(Si)	100% Si($s^{0.67}p^{0.33}$)	1.83	
	LP(N2)	100% N($s^{0.62}p^{0.38}$)	1.98	
	Si1-N1	25% Si($s^{0.39}p^{0.60}d^{0.01}$) + 75% N($s^{0.19}p^{0.80}d^{0.01}$)	1.98	Si: 1.94
	Si2-N1	17% Si($s^{0.10}p^{0.87}d^{0.03}$) + 83% N($s^{0.07}p^{0.92}d^{0.01}$)	1.99	N1: -1.37
	Si1-N2	25% Si($s^{0.39}p^{0.60}d^{0.01}$) + 75% N($s^{0.19}p^{0.80}d^{0.01}$)	1.98	N2: -1.37
	Si2-N2	17% Si($s^{0.10}p^{0.87}d^{0.03}$) + 83% N($s^{0.07}p^{0.92}d^{0.01}$)	1.99	
HNSiNH	N1-H1	70% N($s^{0.32}p^{0.68}$) + 30% H(s)	1.98	
	N2-H2	70% N($s^{0.32}p^{0.68)}$ + 30% H(s)	1.98	
	LP(N1)	100% N($s^{0.43}p^{0.57}$)	1.81	
	LP(N2)	100% N($s^{0.43}p^{0.57}$)	1.81	

All of the data are calculated with the B3LYP hybrid density functional. LP denotes lone pair. natural charges (Q, in e).

Supplementary Table 13. EDA-NOCV results of $H_2Si(\mu\text{-}N)_2SiH_2$ at the meta-Hybrid/M06-2X/TZP level taking $(SiH_2)_2$ and N_2 in the singlet states as interacting fragments. Energy values are in kcal•mol^{-1}.

Energy terms	Orbital interaction	$H_2Si(\mu\text{-}N)_2SiH_2$ ($^1A_1$)
		$(SiH_2)_2$ (^1A) + N_2 ($^1\Sigma_g$)
ΔE_{int}		-547.3
ΔE_{Pauli}		811.4
ΔE_{elstat}[a]		-405.2 (29.8%)
ΔE_{orb}[a]		-953.5 (70.2%)
ΔE_1[b]	$[(SiH_2)_2]$ $^2B_1$ → [NN] $^1A_2$ donation	-329.1 (34.5%）
ΔE_2[b]	$[(SiH_2)_2]$ $^2A_2$ → [NN] $^3B_1$ donation	-318.7 (33.4%)
ΔE_3[b]	$[(SiH_2)_2]$ $^7A_1$← [NN] $^3A_1$ donation	-80.8 (8.5%）
ΔE_4[b]	$[(SiH_2)_2]$ $^7B_2$← [NN] $^1B_2$ donation	-87.5 (9.2%)

[a] The values within the parentheses show the contribution to the total attractive interaction ΔEelstat +ΔEorb. [b] The values within parentheses show the contribution to the total orbital interaction ΔEorb.

Supplementary Table 14. EDA-NOCV results of $H_2Si(\mu\text{-}N)_2SiH_2$ at the meta-Hybrid/M06-2X/TZP level taking $(SiH_2)_2$ and N_2 in the different charged states as interacting fragments. Energy values are in kcal•mol^{-1}.

	$(SiH_2)_2$ (^1A) + N_2 ($^1\Sigma_g$)	$(SiH_2)_2^{2+}$ (^3B) + N_2^{2-} ($^3\Sigma_g$)		$(SiH_2)_2^{4+}$ (^1A) + N_2^{4-} ($^1\Sigma_g$)	$(SiH_2)_2^{6+}$ (^1A) + N_2^{6-} ($^1\Sigma_g$)
ΔE_{int}	-547.3	-1046.1		-2954.8	-6366.1
ΔE_{Pauli}	811.4	792.3		840.0	912.1
ΔE_{elstat}[a]	-405.2(29.8%)	-1142.2(62.1%)		-2937.1(77.4%)	-5492.6(75.5%)
ΔE_{orb}[a]	-953.5(70.2%)	-696.3(37.9%)		-857.7(22.6%)	-1785.6(24.5%)
ΔE_1[b]	-329.1(34.5%）	-110.3(15.8%)	-64.1(9.2%)	-230.7(26.9%)	-712.1(39.9%)
ΔE_2[b]	-318.7 (33.4%)	-100.4(14.4%)	-58.0(8.3%)	-198.3(23.1%)	-311.0(17.4%)
ΔE_3[b]	-80.8(8.5%）	-78.6(11.3%)	-32.7(4.7%)	-107.6(12.5%)	-229.7(12.9%)
ΔE_4[b]	-87.5 (9.2%)	-74.4(10.7%)	-32.4(4.6%)	-97.8(11.4%)	-116.2(6.5%)

Supplementary Methods

Laser-ablated metal atoms reactions with small molecules in excess solid matrix have been introduced in detail[1,2]. In short, a Nd:YAG laser fundamental(1064 nm, 10 Hz repetition rate with 10 ns pulse width and 20−50 mJ/pulse) was focused on a rotating silicon target (Alfa Aesar) generating a bright plume. The laser-ablated silicon atoms reacting with H_2, D_2 (Cambridge Isotopic Laboratories, D, 99.8%), HD (Cambridge Isotopic Laboratories) and H_2 + D_2 mixtures, which were doped in N_2 and $^{15}N_2$ ranged 10%-15%, were condensed at 4K using a closed-cycle helium refrigerator (Sumitomo Heavy Industries Model SRDK-408D2) and reacted for around 2 hours at a rate of 1−2 mmol/h, respectively. Infrared spectra were recorded on a Bruker 80 v spectrometer at 0.5 cm^{-1} resolution between 4000 and 400 cm^{-1} using a HgCdTe range B detector after then. Matrix samples were annealed to a certain temperature for further product formation, usually 7K, and selected samples were subjected to irradiation using LED (light-emitting diode) light (half-wave width 5 nm, 5 W) or a medium-pressure mercury arc lamp (Philips, 175W) ranging from 520 nm to 220 nm wavelength so that more spectra could be recorded.

The Tesla coil discharge of SiH_4 reactions with different concentrations of H_2 in excess solid N_2 were carried out. SiH_4 +N_2 mixtures subjected to Tesla coil discharge in the quartz tube can produce H_2Si. [3]

Post-HF methods (CCSD(T)) and complementary density functional theory (DFT) methods based on hybrid exchanged correlation functionals are preferred owing to their relatively higher computational efficiency and acceptable margin of error in predicting vibration frequencies and zero-point energies[4,5]. Thus, DFT calculations with the B3LYP[6,7] and CCSD(T)[8] density functional with the 6-311++G(3df,3pd) basis[9], aug-cc-pVDZ and aug-cc-pVTZ basis[10,11] were employed with Gaussian 09 program[12]. We found that the bigger basis set does not always give better results, especially for frequency prediction[13-16]. The aug-cc-pVDZ basis set has better performance than aug-cc-pVTZ basis set in the region of 1400-1000 cm^{-1} region[17] and hydrogen containing systems[18,19] for frequency calculation. The orbital composition and partial charges were calculated by a natural bond orbital (NBO) population analysis[12,20]. The bonding analysis of Atoms-in-molecules (AIM)[21], electron localization function (ELF)[22] theory and the multi-center bond order (MCBO), which is also known as multi-center index (MCI) were performed by the Multiwfn code[23]. Nucleus-independent chemical shifts (NICS)[24] were calculated with Gaussian 09 at the same methods and basis[25]. Canonical molecular orbital natural chemical shielding (CMO-NICS) and the resonance structures of compound ***A*** provided by NBO-based Natural Resonance Theory (NRT) analysis were calculated at B3LYP/6-311++G(3df,3pd) level with Gaussian 16 and NBO 6.0 program[26]. The gauge including magnetically induced current (GIMIC) method was calculated at B3LYP/6-311++G(3df,3pd) level with GIMIC2.0 program[27, 28]. The electron density of delocalized bonds (EDDB) were conducted by the RunEDDB program[29, 30].

Supplementary Notes

The N atoms in **A** carry a more negative partial charge of -1.07 e and the Si atom carries a more positive partial charge of 1.45 e (Supplementary Table 12), compared with H_2SiN_2 (0.42 e) and $H_2SiNN(H_2)$ (0.42 e). What's more, *p*-rich (80% or 92%) hybrid orbital of N atoms contributes 75% (or 83%) and *p*-rich (60% or 87%) hybrid orbital of Si with the remaining 25% (or 17%) in Si-N bonding. With the cooperative action of two Si centers in **A**, *p* blocks of Si atoms are more involved in the bond formation, which contributes to the complete activation of N_2.

In the picture of Laplacian distribution of the charge density of **A** in the plane Si-N-N (Supplementary Figure 28), between Si and N, a bond critical point (BCP) found with atoms-in-molecules (AIM) methodology locates in the region with positive Laplacian value and the accumulation of electronic charge, which suggests polar covalent interaction. Between two nitrogen atoms, a BCP may be found without a region of electronic-charge accumulation, which means the two atoms may not be bonded to each other. Supplementary Figure 29 shows the isosurface map of electron localization function (ELF) for **A**. Four trisynaptic basins labeled V (Si, N) with population of 1.97 e in the Si−N interaction region indicate the ionic bonds formed and no covalent interaction between two N atoms based on ELF analysis, which means N≡N bond is broken with the cooperative action of two Si centers in **A**.

Supplementary References

1. Xu, B. et al. Cleavage of the N≡N Triple Bond and Unpredicted Formation of the Cyclic 1,3-Diaza-2,4-Diborete $(FB)_2N_2$ from N_2 and Fluoroborylene BF. *Angew. Chem. Int. Edit.* **60**, 17205-17210 (2021).

2. Xu, B., Shi, P. P., Huang, T. F., Wang, X. F. & Andrews, L. Double and Triple Si-H-M Bridge Bonds: Matrix Infrared Spectra and Theoretical Calculations for Reaction Products of Silane with Ti, Zr, and Hf Atoms. *J Phys Chem A* **121**, 3898-3908 (2017).

3. Andrews, L. & Wang, X. F. Simple ortho-para hydrogen and para-ortho deuterium converter for matrix isolation spectroscopy. *Rev. Sci. Instrum* **75**, 3039-3044 (2004).

4. Wong, M. W. Vibrational frequency prediction using density functional theory. *Chem. Phys. Lett*. **256**, 391-399 (1996).

5. Hadjiivanov, K. I. et al. Power of Infrared and Raman Spectroscopies to Characterize Metal-Organic Frameworks and Investigate Their Interaction with Guest Molecules. *Chem. Rev.* **121**, 1286-1424 (2021).

6. Perdew, J. P. & Wang, Y. Accurate and Simple Analytic Representation of the Electron-Gas Correlation-Energy. *Phys. Rev. B* **45**, 13244-13249 (1992).

7. Becke, A. D. Density-Functional Thermochemistry. Ⅲ. The Role of Exact Exchange. *J. Chem. Phys.* **98**, 5648-5652 (1993).

8. Bartlett, R. J. & Musial, M. Coupled-cluster theory in quantum chemistry. Rev. Mod. Phys. **79**, 291-352 (2007).

9. Frisch, M. J., Pople, J. A. & Binkley, J. S. Self-Consistent Molecular-Orbital Methods .25. Supplementary Functions for Gaussian-Basis Sets. *J. Chem. Phys.* **80**, 3265-3269 (1984).

10. Dunning, T. H. Gaussian-Basis Sets for Use in Correlated Molecular Calculations .1. The Atoms Boron through Neon and Hydrogen. *J. Chem. Phys.* **90**, 1007-1023 (1989).

11. Woon, D. E. & Dunning, T. H. Gaussian-Basis Sets for Use in Correlated Molecular Calculations. Ⅲ. The Atoms Aluminum through Argon. *J. Chem. Phys.* **98**, 1358-1371 (1993).

12. Frisch, M. J.; Trucks, G. W.; Schlegel, H. B.; Scuseria, G. E.; Robb, M. A.; Cheeseman, J. R.; Scalmani, G.; Barone, V.; Mennucci, B.; Petersson, G. A.; et al. *Gaussian 09, revision A.1*; Gaussian, Inc.: Wallingford, CT, 2009.
13. Harabuchi, Y. et al. Anharmonic vibrational computations with a quartic force field for curvilinear coordinates. *J. Chem. Phys.* **151**, 06104 (2019).
14. Samala, N. R. & Jordan, K. D. Comment on a spurious prediction of a non-planar geometry for benzene at the MP2 level of theory. *Chem. Phys. Lett.* **669**, 230-232, (2017).
15. Dobrowolski, J. C. et al. IR low-temperature matrix, X-ray and study on L-isoserine conformations. *Phys. Chem. Chem. Phys.* **12**, 10818-10830 (2010).
16. Dobrowolski, J. C., Jamróz, M. H., Kolos, R., Rode, J. E. & Sadlej, J. IR low-temperature matrix and ab initio study on β-alanine conformers. *Chemphyschem* **9**, 2042-2051 (2008).
17. Sin'ko, S. V., Kuramshina, G. M. & Pentin, Y. A. Quantum-mechanical calculations of the structure and vibrational spectra of $CH_{3-n}Cl_nSiF_3$ (n=0-3) substituted methylsilanes. *Russ. J. Phys. Chem. A* **81**, 917-923 (2007).
18. Nagaraju, M. & Sastry, G. N. Comparative Study on Formamide-Water Complex. *Int. J. Quantum. Chem.* **110**, 1994-2003 (2010).
19. Rode, M. F. & Sadlej, J. The nonadditive effects in the mixed trimers composed of the water dimer and diatomics H_2, HF, HCl, HBr, and ClF. *Chem. Phys. Lett.* **368**, 754-768 (2003).
20. Reed, A. E., Weinstock, R. B. & Weinhold, F. Natural-Population Analysis. *J. Chem. Phys.* **83**, 735-746 (1985).
21. Bader, R. F. W. Atoms in Molecules. *Accounts. Chem. Res.* **18**, 9-15 (1985).
22. Savin, A., Nesper, R., Wengert, S. & Fassler, T. F. ELF: The electron localization function. Angew. Chem. Int. Edit. 36, 1809-1832 (1997).
23. Lu, T. & Chen, F. W. Multiwfn: A multifunctional wavefunction analyzer. *J. Comput. Chem.* **33**, 580-592 (2012).

24. Chen, Z. F., Wannere, C. S., Corminboeuf, C., Puchta, R. & Schleyer, P. V. Nucleus-independent chemical shifts (NICS) as an aromaticity criterion. *Chem. Rev.* **105**, 3842-3888 (2005).
25. Sun, H. C., An, K. & Zhu, J. Triplet State Aromaticity: NICS Criterion, Hyperconjugation, and Charge Effects. *Chem-Asian. J.* **11**, 234-240 (2016)
26. Glendening, E. D., Badenhoop, J. K., Reed, A. E., Carpenter, J. E., Bohmann, J. A., Morales, C. M. & Weinhold, F. NBO,6.0 (Wisconsin Univ., Madison, WI, 2001).
27. Sundholm, D., Fliegl, H. & Berger, R. J. F. Calculations of magnetically induced current densities: theory and applications. *WIREs Comput. Mol. Sci.* **6**, 639-678 (2016).
28. Fliegl, H., Sundholm, D., Taubert, S., Jusélius, J. & Klopper, W. Magnetically Induced Current Densities in Aromatic, Antiaromatic, Homoaromatic, and Nonaromatic Hydrocarbons. *J. Phys. Chem. A* **113**, 8668-8676 (2009).
29. Szczepanik, D. W. et al. A uniform approach to the description of multicenter bonding. *Phys. Chem. Chem. Phys.* **16**, 20514-20523 (2014).
30. Szczepanik, D. W. et al. The electron density of delocalized bonds (EDDB) applied for quantifying aromaticity. *Phys. Chem. Chem. Phys.* **19**, 28970-28981 (2017).
